# Allelic Variation in a Cellulose Synthase Gene (*PtoCesA4*) Associated with Growth and Wood Properties in *Populus tomentosa*

**DOI:** 10.1534/g3.113.007724

**Published:** 2013-11-01

**Authors:** Qingzhang Du, Baohua Xu, Wei Pan, Chenrui Gong, Qingshi Wang, Jiaxing Tian, Bailian Li, Deqiang Zhang

**Affiliations:** * National Engineering Laboratory for Tree Breeding, College of Biological Sciences and Technology, Beijing Forestry University, Beijing 100083, People’s Republic of China; † Key Laboratory of Genetics and Breeding in Forest Trees and Ornamental Plants, Ministry of Education, College of Biological Sciences and Technology, Beijing Forestry University, Beijing 100083, People’s Republic of China; ‡ Department of Forestry, North Carolina State University, Raleigh, North Carolina 27695-8203

**Keywords:** linkage disequilibrium, linkage analysis, multi-locus association models, *Populus tomentosa*, RNA transcript analysis, single nucleotide polymorphism

## Abstract

Lignocellulosic biomass from trees provides a renewable feedstock for biofuels, lumber, pulp, paper, and other uses. Dissecting the mechanism underlying natural variation of the complex traits controlling growth and lignocellulose biosynthesis in trees can enable marker-assisted breeding to improve wood quality and yield. Here, we combined linkage disequilibrium (LD)-based association analysis with traditional linkage analysis to detect the genetic effect of a *Populus tomentosa* cellulose synthase gene, *PtoCesA4*. *PtoCesA4* is strongly expressed in developing xylem and leaves. Nucleotide diversity and LD in *PtoCesA4*, sampled from the *P. tomentosa* natural distribution, revealed that *PtoCesA4* harbors high single nucleotide polymorphism (SNP) diversity (π_T_ = 0.0080 and θ_w_ = 0.0098) and low LD (*r^2^* ≥ 0.1, within 1400 bp), demonstrating that the potential of a candidate-gene-based LD approach in understanding the molecular basis underlying quantitative variation in this species. By combining single SNP, multi-SNP, and haplotype-based associations in an association population of 460 individuals with single SNP linkage analysis in a family-based linkage populations (1200 individuals), we identified three strong associations (false discovery rate *Q* < 0.05) in both populations. These include two nonsynonymous markers (SNP49 associated with α-cellulose content and SNP59 associated with fiber width) and a noncoding marker (SNP18 associated with α-cellulose content). Variation in RNA transcript abundance among genotypic classes of SNP49 was confirmed in these two populations. Therefore, combining different methods allowed us to examine functional *PtoCesA4* allelic variation underlying natural variation in complex quantitative traits related to growth and lignocellulosic biosynthesis.

Wood formation represents a major carbon sink for the biosphere and provides an important renewable resource for lumber, pulp, and paper, and possibly for biofuels feedstocks (Li *et al.* 2006). Wood formation mainly includes deposition of strong secondary cell walls that contain cellulose microfibrils, lignin, and other components. Many studies have examined the molecular biology of secondary cell wall biosynthesis and have shown that the complex, dynamic process of secondary wall formation requires the coordinate regulation of the diverse metabolic pathways involving polysaccharides and lignin (Persson *et al.* 2005; Somerville 2006). As a biomaterial, wood varies in its properties. Specific compositions and structural features make wood more suited for different applications. For example, high-lignin wood can release more thermal energy and thus would be well-suited for thermochemical biofuels applications. Moreover, wood yield is another important trait for trees used as crop species for lumber or biofuels feedstocks. Variation in wood properties likely depends on variation in genes involved in xylogenesis (Neale and Savolainen 2004; Li *et al.* 2009), making these traits amenable to a candidate gene approach. Few functional studies of forest trees have identified genes directly affecting wood quality (Spokevicius *et al.* 2007), largely because of their long generation intervals, large size, and lack of mutant libraries for reverse genetics (González-Martínez *et al.* 2007; Zhang *et al.* 2010a).

Association studies are an effective means to bridge the gap in our understanding between complex quantitative traits and the underlying genetic variation at specific candidate genes or multiple loci dispersed genome-wide (Sexton *et al.* 2012). A diverse group of growth and wood properties has been studied in forest tree species by a candidate gene approach (Thumma *et al.* 2005, 2009; González-Martīnez *et al.* 2007; Wegrzyn *et al.* 2010; Sexton *et al.* 2012; Beaulieu *et al.* 2011; Dillon *et al.* 2010, 2012; Guerra *et al.* 2013). However, linkage disequilibrium (LD) mapping may generate false-positive results because of population structure (Atwell *et al.* 2010), although statistical methods to control for population structure have been developed (Yu *et al.* 2006; Shriner *et al.* 2007). Results from association studies in these species must be cautiously evaluated and, ideally, verified or supported by other approaches, such as quantitative trait locus (QTL) linkage analysis, transgenesis, or transcriptome profiling (Manenti *et al.* 2009; Ingvarsson and Street 2011).

Conventional QTL linkage analysis in controlled crosses and alternative LD-based association mapping using diverse germplasms are two broadly used approaches for the dissection of the genetic architecture of complex traits (Brachi *et al.* 2010; Sterken *et al.* 2012). A linkage approach is powerful for detecting genetic effects at loci involved in the expression of target traits, often identifying large chromosome regions of interest with relatively low marker coverage because QTL mapping uses only the recombination information found in the progeny of two parents. By contrast, LD mapping offers the ability to exploit all recombination events that have occurred in the evolutionary history of a sample set of germplasm, allowing for increased mapping resolution with either previous information on candidate genes or genome-wide scans with very high marker coverage (Manenti *et al.* 2009; Lu *et al.* 2010). The complementary use of traditional linkage mapping and LD-based association mapping would further improve mapping resolution without requiring dense marker maps by combining the advantages and overcoming some of the inherent limitations of both approaches (Myles *et al.* 2009; Lu *et al.* 2010). This integrated strategy enables a closer examination of the number and effect sizes of genes responsible for traits of interest through complex trait dissection in several plant species (Thumma *et al.* 2005, 2009; Stich and Melchinger 2009; Lu *et al.* 2010; Brachi *et al.* 2010). Here, we examined the number and effect magnitudes of allelic polymorphisms in a candidate gene underlying natural variation of growth and wood properties using integrated linkage-LD mapping.

Cellulose is the major component of secondary cell walls; its biosynthesis is catalyzed by cellulose synthases (CesA) located in the plasma membrane (Suzuki *et al.* 2006). The catalytic subunits of the cellulose synthesizing complexes are encoded by the *CesA* gene family, and different sets of *CesAs* dominate cellulose synthesis in primary and secondary cell walls (Taylor *et al.* 2003; Burton *et al.* 2004; Persson *et al.* 2007). For example, *Arabidopsis CesAs* (*AtCesA4*, *AtCesA7*, and *AtCesA8*) are involved in cellulose biosynthesis of the secondary walls (Atanassov *et al.* 2009). Poplars (*Populus* spp.) have a long tradition as a model system for studies of angiosperm tree physiology and genetics. *CesA* homologs have been identified in *Populus*, and they also have been used to investigate the mechanisms of cellulose biosynthesis (Suzuki *et al.* 2006; Kumar *et al.* 2009). The first tree *CesA* gene was isolated from aspen (*P. tremuloides*) by Wu *et al.* (2000). Since then, 17 *CesA* family members have been identified in aspen and its hybrids (*P. tremula* × *tremuloides*) (Djerbi *et al.* 2004). Eighteen *CesAs* (encoding 17 proteins) have been identified in *P. trichocarpa* (Suzuki *et al.* 2006), and five *P*. *trichocarpa CesAs* (*PtiCesA4*, *PtiCesA7-A* and *PtiCesA7-B*, and *PtiCesA8-A* and *PtiCesA8-B*) are expressed in developing xylem tissue undergoing secondary wall thickening (Kumar *et al.* 2009).

Here, candidate gene approaches were used to examine genetic variation in only one of the *Populus tomentosa* cellulose synthase gene homologs, *PtoCesA4*, underlying complex quantitative traits related to growth and lignocellulosic biosynthesis. We used a combination of single SNP models, multi-SNP models, and haplotype-based association methods in an association population (460 individuals) with single SNP linkage analysis in a family-based linkage population (1200 individuals) to identify several associations underlying natural variation of important wood properties. However, to probe the mechanism of this variation, we also examined *PtoCesA4* expression and found that it is expressed in developing xylem and that its expression varies in lines with different genotypes for one of the associated markers.

## Materials and Methods

### Population materials and phenotypic data

#### Association population:

An association population of 460 unrelated *P*. *tomentosa* individuals representing all of the original provenances in the entire natural distribution region of *P*. *tomentosa* (30–40°N, 105–125°E) were used for initial SNP association (Du *et al.* 2013). The distribution zone from which these individuals were collected can be divided into three large climatic regions, southern (S), northwestern (NW), and northeastern (NE), on the basis of a principal components analysis and isodata fuzzy clustering using 16 meteorological factors (Huang 1992). Forty individuals were randomly selected from this association population and used to identify SNPs within *PtoCesA4* using a direct sequencing method.

#### Linkage population:

The hybrid population used for linkage analysis consists of 1200 individuals randomly selected from 5000 F_1_ progeny of controlled crosses between two elite poplar parents (members of the section *Populus*), clone “YX01” (*P*. *alba* × *P. glandulosa*) as the female and clone “LM50” (*P. tomentosa*) as the male. These two related species are members of the section *Populus* in the genus *Populus*. The progeny were grown in 2008 in the Xiao Tangshan horticultural fields of Beijing Forestry University, Beijing, China (40°2′N, 115°50′E) using a randomized complete block design with three clonal replications (Du *et al.* 2012).

#### Phenotypes:

All individuals of these two populations were scored for nine growth and wood property traits, with at least three ramets per genotype. These nine traits included tree height (H), diameter at breast height (D), stem volume (V), fiber length (FL), fiber width (FW), microfiber angle (MFA), holocellulose, α-cellulose, and lignin content. The growth traits, including tree height (H), diameter at breast height (D), and stem volume (V), were measured during field surveys in 2009 using the methods described by Zhang *et al.* (2006). Wood chemical compositions (holocellulose, α-cellulose, and lignin contents) were determined using near-infrared reflectance spectroscopy (NIRS) according to Schimleck *et al.* (2004) based on training sets (models) derived from wet chemistry analyses techniques described in Tian *et al.* (2012). Fiber length and width were measured using the Color CCTV Camera (Panasonic SDII), MFA was measured by X-ray powder diffractometer (Philips, Eindhoven, the Netherlands), and the X-ray diffraction profile was integrated at Chi between −180° and +180°. ANOVA and phenotypic correlations for these nine traits in these two populations were reported by Tian *et al.* (2012) and Du *et al.* (2013), respectively.

### Isolation of *PtoCesA4* cDNA

Developing xylem tissues were collected by scraping the thin (approximately 1.0 mm) and partially lignified layer on the exposed xylem surface at the bottom stems of 1-year-old *P. tomentosa* clone “LM50.” These tissues were immediately frozen in liquid nitrogen and then stored in the laboratory at −80° for later RNA extraction. The *P. tomentosa* stem developing xylem cDNA library was constructed using the Superscript k System (Life Technologies, Rockville, MD) as part of our effort to identify genes expressed predominantly in the *P. tomentosa* stems. The details of constructing the cDNA library were previously described by Li *et al.* (2009). The constructed cDNA library consisted of 5.0×10^6^ pfu, with an insert size range of 1.0–4.0 kb. Random end-sequencing of 1000 cDNA clones and comparison with *Arabidopsis* or *P*. *trichocarpa CesA* sequences identified a full-length cDNA with high similarity to *AtCesA4* (73.6%) or *PtiCesA4* (97.4%). Therefore, we named this cDNA *PtoCesA4*.

### DNA extraction, *PtoCesA4* identification, and phylogenetic analysis

Total genomic DNA was extracted from young leaves with the DNeasy Plant Mini kit (Qiagen China, Shanghai). Specific primers were designed for sequencing *PtoCesA4* based on cDNA sequence; 6421 bp of genomic DNA sequences for *PtoCesA4*, including the promoter (1111 bp), were obtained by direct sequencing in the *P. tomentosa* LM50 clone, using conserved primers, and the BigDye Terminator Cycle Sequencing kit version 3.1 (Applied Biosystems, Beijing, China) run on a Li-Cor 4300 genetic analyzer (Li-Cor Biosciences, Lincoln, NE). The *PtoCesA4* sequence was deposited in GenBank under the accession number KC762249.

To analyze the phylogenetic relationship of *PtoCesA4* to the *CesAs* from other species, the amino acid sequences of CesA from *Arabidopsis thaliana*, rice (*Oryza sativa*), maize (*Zea mays*), *Eucalyptus grandis*, and *Populus trichocarpa* were identified from NCBI (http://www.ncbi.nlm.nih.gov) using BLAST (Altschul *et al.* 1997). Phylogenetic and molecular evolutionary analyses were conducted using MEGA version 4, and the neighbor-joining (NJ) method was used to build phylogenetic trees (Tamura *et al.* 2007). Statistical confidences of the nodes of the tree are based on 1000 bootstrap replicates.

### Tissue-specific expression analysis of *PtoCesA4*

#### RNA extraction and cDNA synthesis:

Total RNA was extracted from various tissues, including root, stem (bark, phloem, cambium, developing xylem, mature xylem), mature leaf, and apical shoot meristem of 2-year-old *P. tomentosa* clone “LM50” with the Qiagen RNAeasy kit (Qiagen China, Shanghai). Additional on-column DNase digestions were performed three times during RNA purification using RNase-Free DNase (Qiagen). RNA was quantified based on absorption at 260 nm. Quantified RNA was reverse-transcribed into cDNA with the SuperScript First-Strand Synthesis system and the supplied polythymidylate primers (Invitrogen). All cDNA samples were used for tissue-specific expression analysis of *PtoCesA4* using real-time quantitative PCR (RT–qPCR).

#### Real-time qPCR:

The qPCR was performed on a DNA Engine Opticon 2 machine (MJ Research) using the LightCycler-FastStar DNA master SYBR Green I kit (Roche). The real-time qPCR and the generated real-time data analysis were performed following the study of Zhang *et al.* (2010a). The *PtoCesA4*-specific (F: 5′-GCCAGTCTGCAACGTCGAA-3′; R: 5′-GGAAAGCCACACACA TGAC-3′) and internal control (*Actin*) primer pairs (F: 5′-CTCCATCATGAAATGCGATG-3′; R: 5′-TTGGGGCTAGTGCTGAGATT-3′) were designed using Primer Express 3.0 (Applied Biosystems). All reactions were performed in triplicate technical and triplicate biological repetitions, respectively. The results obtained for the different tissues were standardized to the levels of *Actin*.

### Transcript analysis of SNP genotypes

Transcript levels were determined for SNP genotypes significantly associated with phenotypic traits to test whether transcript abundance varied in the different SNP genotypic classes. Only significant SNPs [false discovery rate (FDR) *Q* ≤ 0.10] were targeted in the association and linkage populations, respectively (see Results). Transcript levels were determined by RT-qPCR with gene-specific primers. For each SNP genotypic class, 10 trees were individually sampled by obtaining secondary xylem at 1.3 m from the ground; tissue handling and RNA extractions were performed as described. The differential expression across three or two genotypic classes was tested by ANOVA.

### SNP discovery and genotyping

The *PtoCesA4* gene, including 1.111 kb of the promoter, was sequenced and analyzed in 40 unrelated individuals from the *P. tomentosa* association population to identify SNPs without considering insertions/deletions (INDELs). Sequencher v.4.0 and BioEdit were used for sequence alignment, and manual editing was used to confirm sequence quality and to remove primer sequences. To identify putative SNP variants, eight clones for each individual were randomly picked for initial allele sequencing on a Li-Cor 4300 genetic analyzer (Li-Cor Biosciences, Lincoln, NE). Alignments and SNP discovery for this gene among 40 unrelated individuals described here are based on the *P*. *trichocarpa* genome (http://genome.jgi-psf.org/Poptr1/Poptr1.home.html). All 40 sequences have been deposited in GenBank (accession no. KC762252–KC762291). Subsequently, 92 common SNPs (minor allele frequencies > 0.10; see Supporting Information, Table S1) were genotyped by single nucleotide primer extension using a Beckman Coulter sequencing system for all DNA samples.

### Nucleotide diversity and linkage disequilibrium

#### Nucleotide diversity:

Diploid sequences were disambiguated into haplotypes using Phase v.2.1 using 10,000 iterations of the Bayesian Markov chain Monte Carlo chain (Stephens and Scheet 2005). We then used the phased haplotypes to estimate the number of segregating sites, nucleotide diversity, and neutrality test. The DnaSP program version 4.90.1 (Rozas *et al.* 2003) was used to calculate summary statistics for SNP polymorphisms. Nucleotide diversity was estimated using both the average number of pairwise differences per site between sequences, π (Nei 1987), and the average number of segregating sites, θw (Watterson 1975). Diversity statistics were also calculated separately for noncoding, synonymous, and nonsynonymous sites. Neutrality test statistics, Tajima’ D and Fu and Li’s D (Tajima 1989; Fu and Li 1993), were calculated separately for three climatic regions and the complete data set and were tested using 10,000 simulations to test whether a gene or genomic region is evolving randomly (neutral evolution) or whether the region is under selection (non-neutral evolution), and the statistical significance of Tajima’s D was determined using the software DnaSP version 4.90.1.

#### Linkage disequilibrium:

LD was measured as the squared correlation of allele frequencies *r*^2^, which is affected by both recombination and differences in allele frequencies between sites (Hill and Robertson 1968). The *r*^2^ value between each pair of common SNPs (minor allele frequencies > 0.10) in the candidate gene was calculated with 10^5^ permutations using TASSEL v.2.0.1 (http://www.maizegenetics.net/). To assess the extent of LD within the sequenced region of *PtoCesA4*, the decay of LD within a specific physical distance (base pairs) between common SNP sites within this gene was estimated by nonlinear regression (Remington *et al.* 2001). This analysis was performed both within three climatic regions and for the complete data set. Singletons were excluded in the LD analyses.

### Marker–trait association analysis

#### Single-SNP models:

The unified mixed linear model (MLM) was used for single SNP trait analysis, with 10^4^ permutations in TASSEL v.2.0.1 (Yu *et al.* 2006; Bradbury *et al.* 2007). These phenotypes were centered and standardized before analysis. In this MLM (Q + K model) described previously, the population structure matrix (*Q*) was identified based on the significant subpopulation structure in this association population (*K* = 11) (Du *et al.* 2012). The relative kinship matrix (K) has been obtained using the method proposed by Ritland (1996) in Du *et al.* (2013). Corrections for multiple testing were performed using the positive FDR with 10^4^ permutations in QVALUE (Storey and Tibshirani 2003). The modes of gene action were quantified using the ratio of dominance (*d*) to additive (*a*) effects estimated from least-square means for each single SNP association. Details of the algorithm and formulas for calculating gene action were previously described (Eckert *et al.* 2009; Wegrzyn *et al.* 2010).

#### Multi-SNP models:

Bayesian linear mixed models incorporating effects of population structure were used to construct multi-locus models for each trait (Quesada *et al.* 2010; Eckert *et al.* 2012). These phenotypes were centered and standardized before analysis. For each trait, multi-locus models were subsequently constructed from the list of SNPs with significant effects (*P* < 0.05). Model parameters, including 95% credible intervals for SNP effects, were estimated using Markov chain Monte Carlo with 50,000 steps after an initial burn-in of 10,000 steps. All linear mixed-model analyses were conducted using the Bayesian association with the missing data (BAMD) program in R (http://cran.r-project.org/package=BAMD).

#### Haplotype analysis:

On the basis of the information from the LD blocks surrounding the significant SNPs (*P* < 0.05) (see Table S2), the haplotype (a block of linked ordered markers) frequencies of loci genotypes were estimated based on genotypic data of 460 individuals, and haplotype-based association tests with growth and wood quality traits were performed using FAMHAP v.19 (http://famhap.meb.uni-bonn.de/index.html). FAMHAP estimates haplotype frequencies using maximum likelihood. Singleton alleles were ignored when constructing the haplotypes, and haplotypes with a frequency <5% were also discarded. The input consisted of genotype matrices with structure analysis matrices (*Q*) and phenotypic value matrices, and significances of the haplotype associations were identified based on 10^4^ permutation tests. A correction for multiple testing was performed using the positive FDR.

#### Single-SNP linkage analysis:

Comparing the *PtoCesA4* sequences in parents of this linkage population (accession no. KC762249–KC762252), we identified a panel of SNP markers that was based on the common SNPs detected in the association population (see Table S1). Inheritance tests of all SNPs were first examined in the linkage population with 1200 individuals by performing a chi-square (*χ*^2^) test at 0.01 probability, and then SNPs following Mendelian expectations (*P* ≥ 0.01) were used in single-marker analysis in the linkage mapping population (excluding the genotype data involving null alleles at each locus). Significant SNPs were calculated by fitting the data to the model *y* =*µ* + *m_i_* + *e_ij_*, where *y* is the trait value, *µ* is the mean, *m_i_* is the genotype of the *i*th marker, and *e_ij_* is the residual associated with the *j*th individual in the *i*th genotypic class. Percent phenotypic variance explained by the most significant marker was calculated, and the FDR method was used to perform a correction for multiple testing.

## Results

### Identification and phylogenetic analysis of *PtoCesA4*

**Figure 1:**
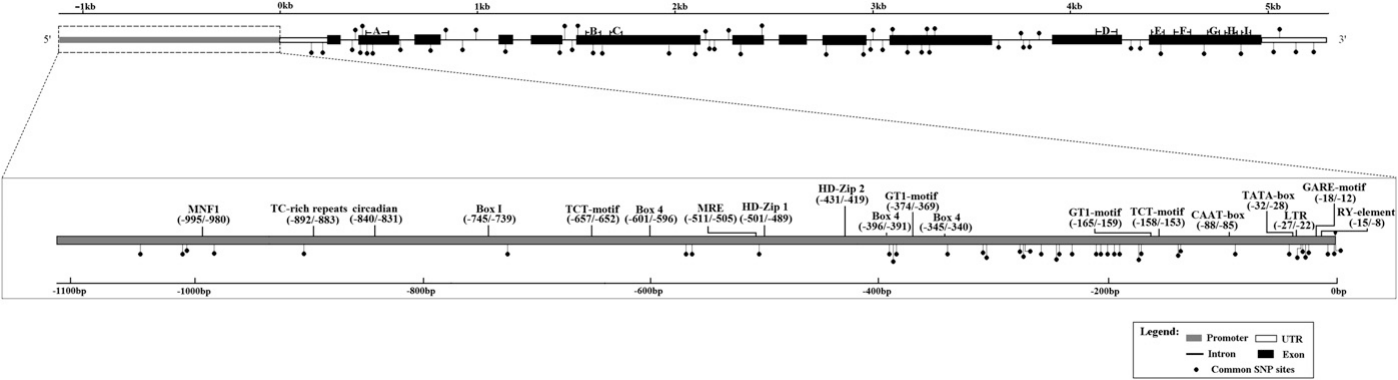
*PtoCesA4* gene structure and the positions of common SNPs (minor allele frequencies > 0.10). All common SNPs are represented by dark spots; putative transcription factor binding sites around SNPs in the *PtoCesA4* promoter were predicted and numbers above the promoter region indicate the positions of putative transcription factor binding sites in base pairs relative to the predicted transcription start site. (A) Zinc-binding domain. (B–I) Two transmembrane helices in the N-terminal region and six in the C-terminal region.

We used reverse-transcription PCR to isolate a full-length cDNA of *PtoCesA4* from a cDNA library prepared from the developing xylem zone of *P*. *tomentosa*. The cDNA clone *PtoCesA4* (GenBank Accession no. KC762292) is 3757 bp in length, and the open reading frame (3129 bp) encodes a polypeptide of 1042 amino acids with an estimated molecular mass of 118.4 kD and a pI of 7.60, flanked by 297 bp of 5′untranslated leader region (5′UTR) and 331 bp of 3′UTR (Figure 1). Nucleotide sequence comparison of *PtoCesA4* cDNAs with known full-length *Arabidopsis CesA* cDNA sequences revealed that *PtoCesA4* is a member of the CesA gene family because it contains all of the conserved features (Holland *et al.* 2000; Chen *et al.* 2010), such as a putative zinc-binding domain (at amino acid residues 31–76), two transmembrane helices (at residues 217–238 and 250–267) in the N-terminal region, and six transmembrane helices in the C-terminal region (Figure 1).

To investigate the evolutionary relationship between the *PtoCesA4* and other eukaryotic *CesAs*, including genes from monocots (rice and maize), Arabidopsis, *Eucalyptus grandis*, and black cottonwood, an unrooted tree was generated from 37 full CesA protein sequences using 1000 replication bootstrap values (see Figure S1). Phylogenetic analysis revealed that the cloned *PtoCesA4* is an ortholog of the *AtCesA4* and *PtiCesA4* (see Figure S1).

### Tissue-specific expression patterns of *PtoCesA4*

**Figure 2:**
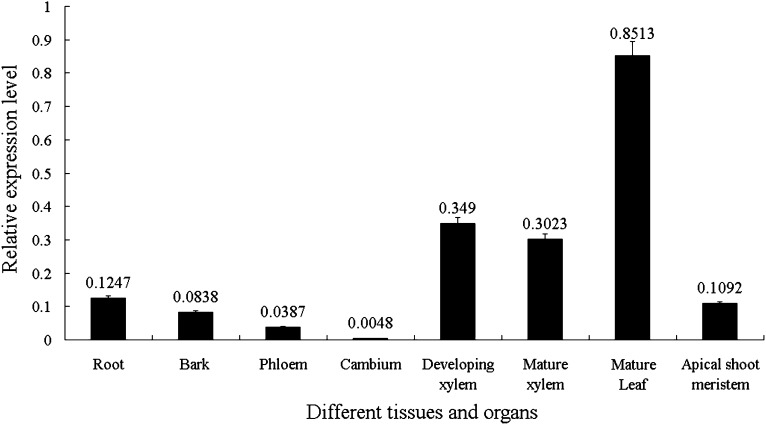
Relative transcript levels of *PtoCesA4* in *Populus tomentosa* tissues and organs. The error bars represent ±SD.

We first determined to what extent *PtoCesA4* exhibited xylem-specific expression. Levels of *PtoCesA4* mRNA in various poplar tissues, including root, bark, phloem, cambium, developing xylem, mature xylem, mature leaf, and apical shoot meristem, wer examined by RT-qPCR with gene-specific primers and *Actin* as an internal control. *PtoCesA4* transcripts were present in all plant organs, including root, stem, and leaf, with varying patterns of expression (Figure 2 and File S1). In leaf and root, *PtoCesA4* is most abundant in mature leaf (0.8513). Low abundance is observed in apical shoot meristem and root (0.1092 and 0.1247). In the stem, *PtoCesA4* shows the highest abundance in the developing xylem tissue (0.3490), followed by the mature xylem (0.3023); it has moderate abundance in the primary tissues of the bark (0.0838) and phloem (0.0387), and the lowest abundance is found in the cambium (0.0048). Collectively, *PtoCesA4* expression in secondary tissues (xylem) was at least 60-fold higher than in primary tissues (cambium) (Figure 2), suggesting that *PtoCesA4* may be a highly expressed gene associated with secondary wall formation. The highest transcript level of this gene was found in the mature leaf (Figure 2), suggesting that it may participate in shared pathways for assimilating the products of photosynthesis into sugars and starch, synthesize cell wall biopolymers, and the create various glycosylated compounds (Persson *et al.* 2005; Geisler-Lee *et al.* 2006).

### Nucleotide diversity in *PtoCesA4*

To characterize the intraspecific molecular evolution of the *PtoCesA4*, an approximately 6421-bp genomic region of *PtoCesA4*, including 1111 bp of promoter region, 297 bp of 5′UTR, 3129 bp of exons, 1553 bp of intron, and 331 bp of 3′UTR, was amplified and sequenced from 40 unrelated individuals in a discovery population that encompassed most of the natural range of *P*. *tomentosa*. After definition of phased haplotypes among these 40 unrelated individuals using Phase v.2.1, a more detailed analysis of SNP variation was conducted over different regions of *PtoCesA4*, and the profile of nucleotide diversity at these loci was calculated (Table 1). On the basis of the aligned sequences for 40 samples, 218 SNPs were detected in *PtoCesA4*, with a high frequency of 1/29 bp (Table 1). The highest level of nucleotide polymorphism was in the promoter region, whereas the lowest was found in the exons, as expected if the coding region is conserved under selective pressure. Fifty-five SNPs were found in exons; of these, eight led to nonsynonymous changes and the other 47 SNPs were synonymous mutations (Table 1). In total, 210 SNPs were categorized as silent sites; 92 of the 218 SNPs (42.2%) were considered common (frequency > 0.10) (Figure 1).

**Table 1:** Nucleotide polymorphism at the *PtoCesA4* locus

Region	Length (bp)	N of Polymorphic Sites	Frequency (bp^−1^)	Transitions and Transversions	Nucleotide Diversity	
					π	θ_w_
Promoter	1111	86	13	1.676	0.0230	0.0246
5′UTR	297	9	33	3.500	0.0053	0.0104
Exon1	51	0	—	0	0	0
Intron1	93	5	19	1.500	0.0060	0.0126
Exon2	202	6	34	0.500	0.0097	0.0105
Intron2	82	6	14	1.000	0.0083	0.0172
Exon3	125	2	63	1.000	0.0047	0.0056
Intron3	299	10	30	1.500	0.0058	0.0080
Exon4	67	1	67	—	0.0072	0.0035
Intron4	96	3	32	—	0.0016	0.0074
Exon5	151	1	151	—	0.0033	0.0016
Intron5	79	6	13	5.000	0.0160	0.0179
Exon6	613	11	56	4.500	0.0042	0.0058
Intron6	173	9	19	8.000	0.0075	0.0164
Exon7	138	3	46	2.000	0.0041	0.0051
Intron7	92	0	—	—	0	0
Exon8	126	4	32	3.000	0.0016	0.0075
Intron8	88	4	22	—	0.0028	0.0134
Exon9	213	5	43	1.500	0.0024	0.0055
Intron9	125	4	31	0.333	0.0061	0.0076
Exon10	510	11	46	10.000	0.0065	0.0065
Intron10	294	8	37	1.667	0.0084	0.0059
Exon11	351	4	88	3.000	0.0008	0.0027
Intron11	132	5	26	4.000	0.0086	0.0089
Exon12	582	7	83	6.000	0.0031	0.0036
3′UTR	331	8	41	1.667	0.0064	0.0057
Total silent*a*	3856.32	210	18	2.060	0.0124	0.0152
Synonymous	714.32	47	15	4.875	0.0149	0.0194
Nonsynonymous	2411.68	8	301	3.000	0.0009	0.0010
Total exon	3129	55	57	2.500	0.0040	0.0048
Total intron	1553	60	26	2.588	0.0065	0.0105
Total*b*	6421	218	29	2.169	0.0080	0.0098

Regions containing indels are excluded from the calculation.

a Total silent indicates synonymous plus noncoding sites.

b Total indicates silent sites plus nonsynonymous sites.

The *PtoCesA4* locus has high nucleotide diversity, where π_T_ = 0.0080 and θ_w_ = 0.0098 (Table 1). The average levels of nucleotide diversity (π) were 0.0124 (silent sites), 0.0040 (exons), and 0.0065 (introns). Both π and θ_w_ were higher in noncoding than in coding regions (Table 1). In coding regions, the average levels of nucleotide diversity for nonsynonymous polymorphisms (*d_N_*, π = 0.0009 and θ_w_ = 0.0010) were approximately six-fold lower than for synonymous polymorphisms (*d_S_*, π = 0.0149 and θ_w_ = 0.0194). The *d_N_*/*d_S_* for the exon regions was significantly less than one, reflecting the action of purifying selection at the nonsynonymous sites in exons. Of the 218 single-base changes, 150 (68.8%) were transitions and 68 (31.2%) were transversions, and the ratio of transitions to transversions for these SNPs was approximately 2.17. Furthermore, for synonymous polymorphisms (47) in exons, 39 (80.1%) were transitions, indicating that translational selection has shaped synonymous codon usage.

Genetic differentiation within and among three geographically independent climatic regions were studied using the nucleotide diversity data from *PtoCesA4* (Table 2). Levels of nucleotide variation (measured using π) in the three climatic regions varied but showed similar patterns of π_tot_, π_sil_, π_s_, and π_n_ (Table 2), suggesting that the level of selective constraint was similar between the climatic regions. Tajima’s D was positive in the southern and northwestern climatic regions but was negative in the northeastern region and in the *P. tomentosa* population as a whole, but no significant departures from the neutral expectation were observed (Table 2). The Fu and Li’s D statistical tests were positive for the northwestern region but were negative for the other regions and the *P. tomentosa* population as a whole (Table 2), revealing an excess of low-frequency mutations for this gene region in the *P. tomentosa* species-wide samples.

**Table 2:** Summary of nucleotide variations for *PtoCesA4* in *Populus tomentosa* natural populations from three climatic regions

Population	N	S	S_xl_	π_tot_	π_sil_	π_s_	π_n_	Tajima’s D	Fu and Li’s D
Northeastern region	14	166	26	0.0074	0.0114	0.0130	0.0008	−0.5013	−0.4495
Southern region	13	149	5	0.0085	0.0131	0.0166	0.0010	0.4973	−0.1296
Northwestern region	13	144	5	0.0082	0.0127	0.0141	0.0010	0.5355	0.0091
Total	40	218	—	0.0080	0.0124	0.0149	0.0009	−0.6498	−2.2043

N, number of sequences sampled; S, number of segregating sites; S_xl_, polymorphic exclusive biallelic mutations in the studied group; π_tot_, average nucleotide diversity in full gene; π_sil_, average nucleotide diversity in synonymous and noncoding sites; π_s_, average nucleotide diversity of synonymous mutation; π_n_, average nucleotide diversity of nonsynonymous mutation.

### Linkage disequilibrium

**Figure 3:**
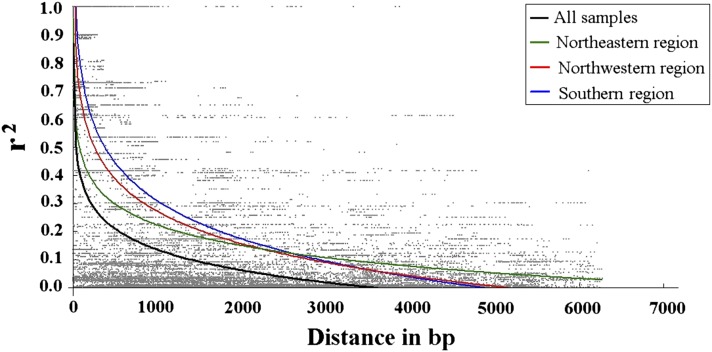
The decay of short-range linkage disequilibrium within *PtoCesA4* for all samples and each climatic region. Pairwise correlations between SNPs are plotted against the physical distance between the SNPs in base pairs. The curves describe the nonlinear regressions of *r*^2^ (Er2) onto the physical distance in base pairs.

Using genotypic data for 92 common SNPs located in *PtoCesA4*, the *r^2^* values were pooled to assess the overall behavior of LD within the *PtoCesA4* gene. The average value of *r^2^* is 0.45 for all SNPs within the *PtoCesA4* region, with a range from 0.0 (equilibrium) to 1.0 (disequilibrium). Several high-LD distinct haplotype blocks (*r^2^* > 0.75; *P* < 0.001) across the sequenced regions were shown. A higher LD level among physically linked loci (the longer haplotype block) is present in the promoter region compared with the other regions (see Figure S2). There are larger numbers of markers that are in linkage equilibrium among these distinct haplotype blocks (*r^2^* < 0.3) (see Figure S2). The nonlinear regression shows a clear and rapid decline of LD with distance in base pairs within *PtoCesA4* (*r^2^* ≥ 0.1 within 1400 bp) (Figure 3), indicating that LD did not extend over the entire gene region. Nevertheless, within-group analyses of LD show a slightly higher level of LD within each geographical climatic region, with the *r*^2^ values declining to 0.1 within approximately 2800 bp (Southern and Northwestern regions) and approximately 3200 bp (Northeastern region) (Figure 3).

### Detection of phenotype-genotype associations

#### Single-SNP–based associations:

A total of 828 tests (92 SNPs × 9 traits, File S2 and File S3) were conducted with 10^4^ permutations using MLM. In all, 41 significant associations with all nine phenotypic traits were identified at the threshold of *P* < 0.05, representing 24 SNPs from different regions within *PtoCesA4* (see Table S2). Corrections for multiple testing using the FDR method reduced these 41 associations to 14 (*Q* < 0.10) (Table 3). These 14 associations representing 10 unique SNPs from the promoter, exon, intron, and 5′UTR regions of *PtoCesA4* were significantly associated with seven phenotypic traits, excluding fiber length and microfibril angle traits (Table 3). These loci explained a small proportion of the phenotypic variance, ranging from 1.6% to 5.3% (Table 3), in accordance with polygenic quantitative models of wood traits (Neale and Savolainen 2004; Beaulieu *et al.* 2011).

**Table 3:** Summary of significant SNP marker–trait pairs identified in the *Populus tomentosa* association population using the mixed linear model after a correction for multiple testing

Trait	Locus	Position	Mutation	Fst	Association Population (*N* = 460)			2*a**a*	*d**b*	*d*/*a*	2*a*/s_p_*c*	Frequency*d*	*a**e*
					*P*-value	*Q*-value	*R*^2^ (%)						
Lignin													
	SNP44	Intron1	[G: T]	0.057	0.0012	0.0551	2.1	3.42	−0.98	−0.5725	3.8320	0.15 (T)	0.8880
	SNP49	Exon 3	[C: A]^ns^	0.114	0.0025	0.0810	3.0	1.58	−0.35	−0.4381	1.7746	0.14 (A)	−0.387
α-Cellulose													
	SNP3	Promoter	[G: A]	0.077	0.0015	0.0629	2.2	4.79	0.84	0.3512	1.8635	0.11 (A)	2.7436
	SNP18	Promoter	[A: T]	0.039	0.0011	0.0551	2.5	0.44	1.81	8.1825	0.1720	0.45 (T)	−0.1896
	SNP41	5′UTR	[C: T]	0.100	3.02E-05	0.0035	1.6	0.31	−0.60	−3.8387	0.1206	0.17 (T)	0.5131
	SNP49	Exon 3	[C: A]^ns^	0.114	0.0031	0.0948	5.3	2.02	−0.19	−0.1881	0.7857	0.14 (A)	0.0466
Holocellulose													
	SNP45	Exon 2	[C: A]^s^	0.050	0.0002	0.0142	4.0	1.31	0.61	0.9309	0.3429	0.16 (A)	0.8728
	SNP81	Intron 10	[T: C]	0.052	0.0002	0.0142	3.0	0.61	0.06	0.1803	0.1597	0.46 (C)	−0.0412
Fiber width													
	SNP59	Exon 6	[A: C]^ns^	0.130	0.0008	0.0440	2.6	0.71	−0.12	−0.3426	0.3567	0.38 (A)	−0.2106
Diameter at breast height (D)													
	SNP48	Intron 2	[A: T]	0.091	0.0009	0.0454	1.9	2.12	−3.05	−2.8770	0.3695	0.44 (T)	−0.1966
	SNP75	Exon 10	[T: C]^s^	0.036	3.15E-05	0.0035	3.2	0.83	0.13	0.3206	0.1452	0.43 (C)	0.2125
	SNP81	Intron 10	[T: C]	0.052	0.0003	0.0195	2.0	2.05	0.14	0.1388	0.3567	0.46 (C)	−0.5694
Tree height (H)													
	SNP49	Exon 3	[C: A]^ns^	0.114	0.0012	0.0551	2.3	0.89	−0.09	−0.191	0.3053	0.14 (A)	0.0198
Stem volume (V)													
	SNP75	Exon 10	[T: C]^s^	0.036	3.02E-05	0.0035	2.6	0.06	0.01	0.1453	0.1479	0.43 (C)	0.0164

Fst indicates variation attributable to differentiation among subpopulations. *R^2^* indicates percentage of the phenotypic variance explained. *P*-value indicates significance level for association (significance is *P* ≤ 0.05). *Q*-value indicates a correction for multiple testing (false discovery rate (*Q*) ≤ 0.10). ns, nonsynonymous polymorphism; s, synonymous polymorphism.

a Calculated as the difference between the phenotypic means observed within each homozygous class (2a = |G_BB_-G_bb_|, where G_ij_ is the trait mean in the *ij*th genotypic class).

b Calculated as the difference between the phenotypic mean observed within the heterozygous class and the average phenotypic mean across both homozygous classes [d = G_Bb_− 0.5(G_BB_+ G_bb_), where G_ij_ is the trait mean in the *ij*th genotypic class].

c s_p_, SD for the phenotypic trait under consideration.

d Allele frequency of either the derived or the minor allele. Single nucleotide polymorphism (SNP) alleles corresponding to the frequency listed are given in parentheses.

e The additive effect was calculated as a = p_B_(G_BB_) + p_b_(G_Bb_) − G, where G is the overall trait mean, G_ij_ is the trait mean in the ij^th^ genotypic class, and p_i_ is the frequency of the i^th^ marker allele. These values were always calculated with respect to the minor allele.

**Figure 4:**
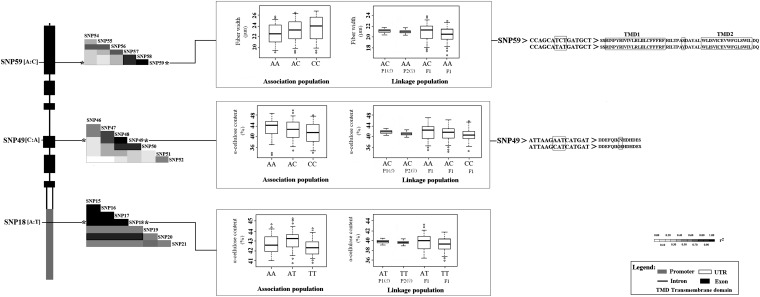
Genotypic effects of the significant single nucleotide polymorphisms (SNPs) in *PtoCesA4* on the same phenotypic trait in association and linkage populations. The marker SNP49 in exon 3 of *PtoCesA4*, a nonsynonymous mutation, which results in an encoded amino acid change from His to Asn, was significantly associated with α-cellulose content in association and linkage populations. The AA homozygotes were associated with higher α-cellulose values and CC homozygotes were associated with lower α-cellulose values, and mean values in AC heterozygotes were medium in both populations, which are supported by the observation that SNP49 has an additive effect on gene action in cellulose content. The nonsynonymous marker SNP59 in exon 6 of *PtoCesA4* significantly associated with fiber width in both populations and shows patterns of gene action consistent with additive effects on fiber width. The A allele at SNP59 causes a Ser-to-Tyr amino acid substitution (d) SNP18 from the promoter of *PtoCesA4* and showed significant association with α-cellulose content in both populations. The differences in α-cellulose content among the three genotypes of this marker indicate that patterns of gene action are consistent with overdominance effects. P1 represents the female clone YX01 (*Populus alba* × *Populus glandulosa*), P2 represents the male clone LM 50 (*Populus tomentosa*), and F1 represents the hybrid progeny.

Of these 10 unique SNPs, there were two nonsynonymous, two synonymous, and six noncoding SNPs (Table 3). The nonsynonymous marker SNP49 in exon 3 results in an amino acid change from His to Asn, associated significantly with multiple traits, *i.e.*, α-cellulose (5.3%), lignin (3.0%), and H (2.3%). In this case, the mode of gene action seems additive, with the minor allele (A) conferring a lower lignin content and higher values in α-cellulose and H (Table 3). For the other nonsynonymous marker, SNP59 in exon 6, which has the minor allele (A), results in an amino acid change from Ser to Tyr, associated significantly with fiber width (*R^2^* = 2.6%). The genotypic effects on fiber width were significant (22.38 µm in AA, 23.21 µm for AC, and 23.99 µm for CC), consistent with the additive effect of gene action on fiber width (Table 3 and Figure 4). Also, a synonymous marker SNP45 in exon 2, associated with holocellulose content, showed a difference among three genotypic classes (two significant) (74.62% in AA, 74.55% in AC, and 73.30% in CC), indicating patterns of gene action consistent with dominant effects (Table 3). SNP75 in exon 10, the other synonymous mutation, associated with D and V, explaining 3.2% and 2.6% of the phenotypic variance, respectively (Table 3).

Of the remaining noncoding markers, SNP3 and SNP18 from the promoter region and SNP41 from the 5′UTR were significantly associated with α-cellulose content, with the small single SNP effects ranging from 1.6% to 2.5% (Table 3). SNP81 in intron 10 was significantly associated with holocellulose and D traits, and this marker has the same allelic effects in these two traits: heterozygous trees (CT) for this marker showed intermediate average holocellulose content (73.78% in CT *vs.* 74.03% and 73.42% in CC and TT, respectively). An additive effect of gene action appeared in the D trait (20.26 cm in CC, 21.43 cm in CT and 22.31 cm in TT). SNP44 in intron 1 and SNP48 in intron 2 were associated with lignin content and D, respectively. Four of the 10 SNP markers exhibited significant associations with at least one trait, suggesting a pleiotropic effect of these loci (Table 2).

#### Multi-SNP associations:

**Figure 5:**
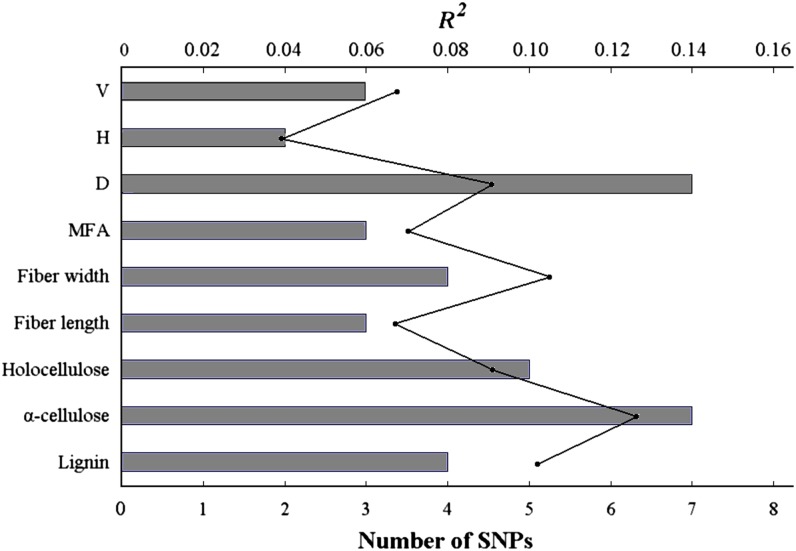
Multi-locus single nucleotide polymorphism (SNP) models explain a large percentage of the phenotypic variance for growth and wood properties in the *Populus tomentosa* association population. The gray line and points denote the numbers of SNPs identified for each trait and the marker effects (*R^2^*) explained by the list of SNPs identified using the Bayesian mixed linear model in the Bayesian association with missing data (BAMD) program in R (http://cran.r-project.org/package=BAMD).

Application of Bayesian linear mixed models, in which each trait was evaluated against multi-SNP models, identified a multitude of new genetic associations (see Table S3). In total, 38 associations were obtained across all traits representing nine unique growth and wood properties and 22 unique SNPs. Of the 22 unique SNPs, three were nonsynonymous, four were synonymous, and 15 were noncoding (see Table S3). Ten of these SNPs were associated with more than one trait (range, 1–4), which is likely to be attributable to the strong correlation between some wood and growth property traits. Effect sizes for SNPs identified with the multi-locus models when analyzed using a single locus test were nearly two-fold lower than those detected only in the single-marker models (average *R^2^* = 0.015). All of the SNPs identified as significant in the single locus tests (FDR *Q* < 0.10) were revealed with significant effects in multi-SNP models (Table 3). The number of SNPs retained in these models ranged from two (H) to seven (α-cellulose and D), with a mean of four SNPs per trait, and explained larger portions of genetic effects for many traits ranging from 3.9% to 12.4% (Figure 5).

#### Haplotype-based associations:

In this low-LD tree species, haplotype-based association tests were performed to identify significant haplotypes with growth and wood quality traits (Table 4). Sixty-eight sets (based on the significant single SNPs at the threshold of *P* < 0.05) (see Table S2) were analyzed with each of the nine traits, and the number of common haplotypes (frequency > 1%) per set varied from 2 to 10, with an average of 5.0. Seventeen significant regions including 80 common haplotypes were identified at the significance threshold of *P* < 0.05 (details not shown). Multiple test corrections reduced this number to 11, at a significance threshold of *Q* < 0.10, and 21 significant haplotypes were associated with the five phenotypic traits, excluding MFA and H phenotypes (Table 4).

**Table 4:** List of haplotypes with significant associations with wood quality and growth traits in the *P. tomentosa* association population (n = 460) after a correction for multiple testing

Trait	*P*-value	*Q*-value	*R^2^*(%)	Significant Haplotypes	Haplotype Frequency	Single-Marker Associations*a*
Lignin						
	0.0012	0.0487	3.4	SNPs 2-4		—
				T-G-T	0.28	
				G-A-C	0.18	
	0.0052	0.0760	3.7	SNPs 44-46		SNP44 (lignin, *Q* = 0.0551)
				G-A-T	0.05	
				T-A-T	0.27	
α-Cellulose						
	0.0019	0.0532	3.8	SNPs 1-3		SNP3 (α-cellulose, *Q* = 0.0629)
				T-G-A	0.25	
				T-T-G	0.43	
	0.0015	0.0487	2.8	SNPs 16-18		SNP18 (α-cellulose, *Q* = 0.0551)
				T-A-A	0.08	
	0.0063	0.0922	5.6	SNPs 48-50		SNP49 (α-cellulose, *Q* = 0.0948)
				T-A-C	0.16	
				T-C-A	0.17	
Holocellulose						
	0.0040	0.0713	3.0	SNPs 38-40		—
				T-G-A	0.12	
	0.0023	0.0579	5.1	SNPs 44-46		SNP45 (holocellulose, *Q* = 0.0142)
				T-A-T	0.09	
				G-C-C	0.13	
				G-A-C	0.20	
Fiber length	0.0051	0.0760	4.0	SNPs 89-91		—
				C-A-A	0.05	
				G-G-A	0.29	
Fiber width	0.0022	0.0579	3.2	SNPs 57-59		SNP59 (fiber width, *Q* = 0.0440)
				C-T-A	0.05	
D						
	0.0005	0.0187	2.7	SNPs 47-49		SNP48 (D, *Q* = 0.0454)
				C-A-C	0.21	
				T-T-A	0.17	
	0.0035	0.0673	2.6	SNPs 81-83		SNP81 (D, *Q* = 0.0454)
				C-T-A	0.30	
V	0.0030	0.0611	3.9	SNPs 75-77		SNP75 (V, *Q* = 0.0035)
				T-T-A	0.11	
				C-T-G	0.08	

*R^2^* indicates percentage of the phenotypic variance explained. *P*-value indicates the significant level for haplotype-based association (the significance is *P* ≤ 0.05). *Q*-value indicates a correction for multiple testing (false discovery rate (Q) ≤ 0.10). D, diameter at breast height; V, stem volume.

a Significant single-marker associations with the lowest *Q* value (FDR *Q* ≤ 0.10) relating to the significant haplotype–trait association; /, no data were identified in this study.

Most of significantly haplotype-based associations were trait-specific, but some were shared among the traits. For instance, several haplotypes from SNP44 to SNP46 were simultaneously associated with lignin and holocellulose, which are supported by significant single SNP associations (*Q* < 0.10) (Table 3 and Table 4). SNP89 to SNP91, in the 3′UTR region, were associated with fiber length without a supporting single marker association (Table 4), and significant differences among haplotypes were observed for this trait (1.180 mm in G-G-A and 1.501 mm in C-A-A). Each haplotype explained a small proportion of phenotypic variation, from 2.6% to 5.6%, and many were strongly supported by single-SNP associations (Table 3 and Table 4).

#### Single-SNP linkage analysis:

Based on all 92 common SNPs detected by association mapping we observed 56 SNPs from the *PtoCesA4* in the linkage population, including six novel alternative SNPs identified in the parents of this hybrid population and 50 corresponding to the positions of common SNPs detected in the association population (see Table S1). Of these SNPs, 46 markers segregated in the 1200 progeny, with a segregation ratio close to 1:2:1 for 26 SNP loci and 1:1 for 20 loci (see Table S1); the 10 significant SNP markers (*Q* < 0.10) (Table 3) identified in the association population were involved in this single-SNP linkage analysis. Therefore, 414 single-marker analyses (46 SNPs × 9 traits) (see File S4 and File S5) were conducted in this linkage mapping population. In all, 18 associations were first observed at the threshold of *P* < 0.05 (Table S4). However, a multiple test correction reduced this number to seven (*Q* < 0.10) (Table 5). Of these, three significant SNP markers were associated with α-cellulose content (SNP18, SNP49, and SNP75); one SNP marker each was associated with holocellulose, fiber length, and fiber width, and H traits were observed in the linkage population, with marker effects that varied from 1.5 to 3.6% (*Q* < 0.10) (Table 5).

**Table 5:** Summary of significant SNP marker–trait pairs identified in *PtoCesA*4, using a linkage population, after correction for multiple testing errors

Trait	Locus	Position	Alleles of Parents (Female: Male)	Linkage Population (*N* = 1200)		
				*P*-value (*P* ≤ 0.05)	*Q*-value (*Q <* 0.10)	*R*^2^ (%)
α-Cellulose	SNP18	Promoter	[TT: AT]	0.0036	0.0693	2.8
	SNP49	Exon 3	[AC: AC]	0.0015	0.0490	3.6
	SNP75	Exon 10	[CT: CT]	0.0019	0.0532	1.5
Holocellulose	SNP88	Exon 12	[AG: AG]	0.0034	0.0693	1.9
Fiber length	SNP70	Intron 9	[AT: AT]	0.0013	0.0490	3.0
Fiber width	SNP59	Exon 6	[AA: AC]	0.0044	0.0693	2.5
Tree height (H)	SNP51	Intron3	[AC: AC]	2.55E-05	0.0050	3.0

*R^2^* indicates percentage of the phenotypic variance explained. *P*-value indicates significance level for association (significance is *P* ≤ 0.05). *Q*-value indicates a correction for multiple testing (false discovery rate (*Q*) ≤ 0.10).

SNP18 and SNP49 with α-cellulose and SNP59 with fiber width were identified in both association and linkage populations (Table 3 and Table 5). Because SNP49 was heterozygous in both parents (AC: AC) of the family-based linkage population, the effects of different genotype classes (AA, AC, and CC) at SNP49 for α-cellulose content were similar in both populations (Figure 4), which are supported by the observation that SNP49 has an additive effect on cellulose content (Table 3 and Figure 4). Similarly, for the noncoding marker SNP18, the heterozygous trees (AT) showed higher average α-cellulose content than the homozygous trees TT (40.18% in AT and 39.20% in TT; significant), indicating that the minor allele T was dominant, and genotypic effects on α-cellulose content were consistent in both populations (Figure 4). In the linkage population, the SNP59 genotype was different in the parents (AA: AC), and the significant effects among the corresponding genotypic classes in SNP59 (20.29 µm in AA, 21.40 µm in AC) were consistent in the association population (Table 3 and Table 5). Further analysis of the data suggested that these single markers also supported the haplotype-based associations with corresponding traits (Table 4 and Table 5).

### Transcript analysis of SNP genotypes

**Figure 6:**
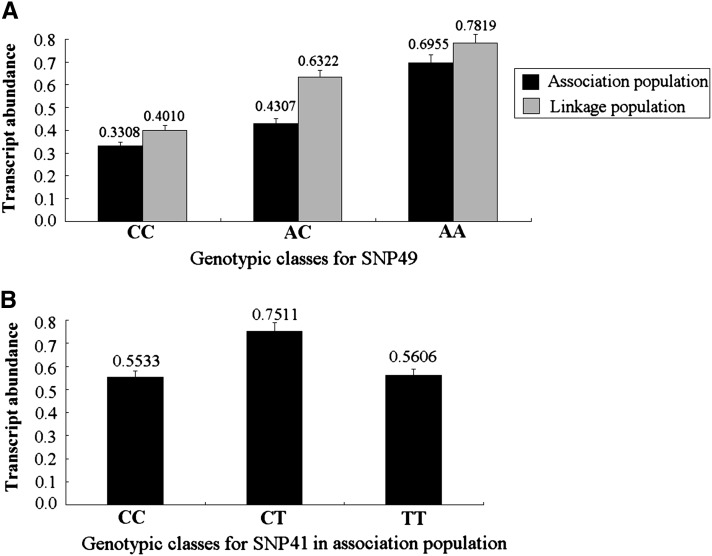
*PtoCesA4* transcript abundance varies among genotypic classes for significant SNP associations. (A) Transcript abundance variation of three genotypic classes for SNP49 in both association and linkage populations. The black and gray lines represent the transcript levels among three genotypic classes in association and linkage populations, respectively. (B) The relative mRNA transcript levels of *PtoCesA4* among three genotypic classes for SNP41, a significant noncoding marker in the 5′UTR region of *PtoCesA4*. The error bars represent ±SD.

To determine whether these significant allelic SNPs affect the *PtoCesA4* RNA transcript abundance, transcript levels were compared among the different genotypic classes for the 10 significant SNPs (*Q* < 0.10, Table 3) in the association population and seven (*Q* < 0.10) (Table 5) in the linkage population using RT–qPCR with gene-specific primers. Measurement of differential transcript abundance across three or two genotypic classes (10 trees for each genotype) for each of the 17 SNPs indicated that two markers (SNP41 and 49) exhibited significant differences in the RNA transcript levels among the three genotypes in the association population, but only SNP49 was detected in the linkage population (Figure 6). The genotypic abundance ratio estimates for these SNPs had very low SEs, suggesting that these estimates are robust (Figure 6).

In the association population, for the marker SNP49 (exonic) associated with three traits, the highest transcript abundance was found in the AA group (0.6955), followed by the AC group (0.4307), and the transcript levels of the CC group were lowest (0.3308) (Figure 6A). In examining genotype-specific transcript levels for SNP41 (5′UTR), the heterozygous trees (0.7511 in CT) for this marker showed higher relative abundance than the homozygous trees, and the transcript level differences between SNP homozygous trees were not significant (0.5533 in CC and 0.5606 in TT) (Figure 6B). In the linkage population, SNP49 also showed a significant difference in transcript level among three distinct genotype classes, and the transcript levels for the groups AA, AC, and CC were 0.7819, 0.6322, and 0.4010, respectively (Figure 6A). These differences in transcript abundance among genotypic classes are discussed regarding their putative function and relationship with variation in phenotypic trait.

## Discussion

*PtoCesA4*, a highly targeted candidate gene belonging to key pathways of secondary cell wall biosynthesis, was investigated using a more comprehensive approach than previous studies of trees (Wegrzyn *et al.* 2010; Sexton *et al.* 2012; Beaulieu *et al.* 2011; Dillon *et al.* 2010, 2012; Guerra *et al.* 2013). Phylogenetic analysis of *PtoCesA4* and other eukaryotic *CesA*s in land plant lineages revealed that *PtoCesA4* is an ortholog of *AtCesA4* and *PtiCesA4*. Functional studies of these putative orthologs informed the structure of *PtoCesA4* and its allelic diversity affecting complex traits controlling growth and lignocellulosic biosynthesis in *P. tomentosa*. Given the rapid decay of within-gene linkage disequilibrium in *Populus*, it is better to extend SNP discovery across the full-length gene sequence, including promoter regions, to avoid missing important allelic polymorphisms. Several small-effect single-SNP or haplotype-based associations were detected in this species, indicating wood properties are quantitative traits controlled by multiple alleles, and limited statistical power for single-marker or haplotype-based association methods (Beaulieu *et al.* 2011; Du *et al.* 2013). Therefore, using linkage-LD mapping approaches combined with transcriptomic comparison of genotypic classes of each significant SNP allowed us to examine functional *PtoCesA4* allelic variation responsible for complex quantitative traits related to lignocellulosic biosynthesis in trees.

### Nucleotide diversity and LD in *PtoCesA4*

Detailed knowledge of levels of nucleotide diversity and the extent of LD in natural populations is important for understanding the forces responsible for evolutionary change and for evaluating the precision and power of association mapping (Ingvarsson 2008a; Zhang *et al.* 2010b). As a prerequisite for SNP-based association mapping, a comprehensive investigation of the patterns of SNP distribution and frequency within the full-length *PtoCesA4* locus and among natural populations of *P. tomentosa* was required. Levels of average nucleotide diversity in coding regions were substantially lower than in the noncoding regions (Table 1), reflecting that the coding regions are conserved relative to the other regions under natural pressure. Within coding regions, the π_nonsyn_:π_syn_ ratio (0.0604) was significantly less than 1 for *PtoCesA4*, which was commonly observed in natural populations of forest trees (Krutovsky and Neale 2005; González-Martínez *et al.* 2007; Ingvarsson 2008a). Synonymous mutations occurring during evolution may be fixed with a higher probability than neutral ones because of purifying selection (Zhang *et al.* 2010b). Furthermore, an excess of transitional over transversional substitutions was found in this gene. There is a universal bias in favor of transitions over transversions, possibly as a result of the underlying chemistry of mutation, such as the relatively high rate of mutation of methylated cytosines to thymine or, particularly, the selection for codon usage bias in coding regions (Keller *et al.* 2007; Ingvarsson 2008b). For synonymous polymorphism (47) in *PtoCesA4*, 80.1% (39) were transition substitutions, indicating that translational selection has shaped synonymous codon usage. This finding was supported by the most common model of synonymous codon usage indicating that one or a few synonymous codons are preferentially used in genes with high codon bias, and such codons usually end in C or G (Ingvarsson 2008b). Moreover, the intron region harbored significantly less nucleotide diversity than synonymous sites (Table 1), similar to patterns recently reported for *P. balsamifera* (Olson *et al.* 2010) and *Medicago truncatula* (Branca *et al.* 2011). Introns experience higher selective constraints than synonymous coding sites, possibly because introns harbor key regions for regulation of expression (Wray *et al.* 2003; Thumma *et al.* 2005). Surveys of SNPs diversity in genes have been mainly focused on exons, introns, and UTRs, with less attention to promoter regions. It will be of interest to compare the level of nucleotide diversity within the promoters with a much larger diverse survey of the other gene regions. In this study, a significantly higher frequency of polymorphisms was found in the promoter region than in the other regions in *PtoCesA4* (π_T_ = 0.0230) (Table 1). This finding suggests that this regulatory region may be relatively unstable and a “hotspot” for genetic change (Weickert *et al.* 2012).

In this study, the level of LD decay in *PtoCesA4* was analyzed separately within each of the three climatic regions and for the complete natural population (Figure 3), and results showed that northwestern and southern regions seem to have experienced similar histories. The northeastern region had higher LD than the northwestern and southern regions, consistent with the higher frequency of exclusive SNPs observed in this region (Table 2). The fine-scale LD pattern among polymorphic SNP markers in candidate genes may be influenced by gene conversion in different sampling populations. Generally, low LD might result from a species-wide scale of sampling, which incorporates the entire history of polymorphism and recombination over thousands of generations (Morrell *et al.* 2005; Kim *et al.* 2007). Our results support this sample-scale explanation, which shows that the LD in *PtoCesA4* within three climatic regions may be more extensive than the LD found in our range-wide *P. tomentosa* samples (Figure 3), consistent with previous studies (Olson *et al.* 2010; Branca *et al.* 2011). However, a recent genome-wide study of the extensive LD in *P. trichocarpa* (*r^2^* > 0.2, within 3–6 kb) suggests that genome-wide association studies and genomic selection in natural populations may be more feasible in *Populus* than previously assumed (Slavov *et al.* 2012). Therefore, our future work will focus on estimation of LD decay with greater genomic coverage and exploration of the variability of haplotype structure across the entire genome. Such studies also will help to elucidate how *Populus* managed to adapt to a wide variety of environmental conditions (Ingvarsson and Street 2011).

### Dissecting allelic polymorphisms underlying growth and wood properties

Estimating population structure is an important prerequisite in LD-based association analysis, and it is important to avoid false-positive results or spurious associations and to constrain association studies in association populations (Du *et al.* 2012). MLM methods have proven useful in controlling for population structure and individual relatedness within association mapping studies, with the population structure matrix (*Q*) and the relative kinship matrix (K) as the covariance. In this study, the observed effect of the population structure (when K = 11 or K = 3) (Du *et al.* 2012) on phenotypic variation is not significant, and the number and power of significant associations identified in two settings are stable (data not shown), suggesting that tree species are ideal for the fine-mapping of candidate genes and functional analysis of gene variants, because they are predominantly outcrossing, and have large, effective, relatively unstructured population sizes.

The variation in quantity and quality of primary and secondary wall cellulose in plants is suggested to be the result of enzymatic activities of different types of cellulose synthase (CesA) (Somerville 2006). Secondary cell walls have a higher percentage of cellulose, a higher degree of polymerization, and a higher crystallinity than xylem primary walls (Joshi *et al.* 2004). Because of the importance of secondary walls in determining wood quality traits, many researchers have focused on secondary cell wall *CesAs*. In this study, *PtoCesA4* was originally isolated from a developing xylem cDNA library of *P. tomentosa* and was found to have xylem-specific expression patterns (Figure 2). Similarly, its putative ortholog in Arabidopsis (*AtCesA4*) was specifically associated with secondary cell wall development (Atanassov *et al.* 2009), and the ortholog in *P. trichocarpa* (*PtiCesA4*) also is expressed in developing xylem tissue undergoing secondary wall thickening (Suzuki *et al.* 2006). In addition to the direct coding of CesA subunit proteins, genetic evidence has confirmed an effect of *CesA* on wood chemical properties, influencing cellulose/hemicellulose content as well as lignin content and composition (Song *et al.* 2010; Wegrzyn *et al.* 2010). Several candidate genes in other pathways are also involved in synthesizing cellulose (Szyjanowicz *et al.* 2004; Coleman *et al.* 2009). On the basis of these studies, we dissected allelic polymorphisms within *PtoCesA4*, underlying growth and wood properties, by using LD-based association in *P. tomentosa*, combined with single-SNP linkage analysis. Because of the low LD in *P. tomentosa* (Figure 3), once a marker–trait association has been discovered and validated, it is likely that such a marker is located in close proximity to the causal polymorphisms or even the functional variant itself (Neale and Kremer 2011). Two nonsynonymous markers (SNP49 and SNP59) and a noncoding marker (SNP18) were associated with the same traits in both the association and linkage populations (Table 3 and Table 5), confirming the value of an integrated approach for characterizing the genetic basis of wood traits. We also found that the population differentiation (*Fst* = 0.075) (Table 3) for these significant SNPs was greater than that (*Fst* = 0.028) of all common SNPs identified in the association population, which is consistent with the report that putative functional SNPs in genes in etiologic pathways for CVD show greater population differentiation than nonfunctional SNPs (Kullo and Ding 2007).

Using single marker association, a nonsynonymous substitution in exon3 of *PtoCesA4* (SNP49) was in strong association with multiple traits (α-cellulose, lignin, and H), and the modes of gene action appeared to be additive, with the minor allele (A) conferring a lower lignin content and higher values in α-cellulose and H. This marker also was identified with these three traits in the multi-SNP analysis (see Table S3). This is consistent with the significant phenotypic correlation between these three traits (Du *et al.* 2013) and also represents a pleiotropic effect of *PtoCesA4* on certain traits. Wood is composed of cellulose microfibrils embedded in a lignin–hemicellulose matrix. The observed associations of *PtoCesA4* with diverse traits suggest that *PtoCesA4* influences two distinct pathways (lignin and cellulose biosynthesis) in secondary cell wall synthesis (Song *et al.* 2010; Du *et al.* 2013). A similar phenomenon has been identified in previous studies (Thumma *et al.* 2009; Wegrzyn *et al.* 2010). Cellulose biosynthesis is coexpressed with other biological processes in plant vascular development, and the genes involved in these shared pathways often are functional homologs (Somerville *et al.* 2004; Eckert *et al.* 2012). For instance, genes encoding lignin monomer-polymerizing laccases and lignin monomer synthesis enzymes are among the most closely coexpressed genes with secondary cell wall *AtCesA4*, *AtCes7*, and *AtCes8* (Persson *et al.* 2005). The inverse genotype effects in SNP49 between α-cellulose and lignin content might be indirectly related to carbon distribution toward the synthesis of C5 or C6 sugars (Guerra *et al.* 2013), which is in accordance with the significant negative phenotypic correlation between α-cellulose and lignin content in both *P. tomentosa* association and linkage populations (Du *et al.* 2013). Moreover, common haplotypes (SNPs48–SNP50) associated with α-cellulose traits surround SNP49, and this locus was identified in the multi-SNP associations (Table 4). We also observed a significant association between SNP49 and α-cellulose in the linkage population with the same genotypic effect for this locus in association populations (Table 5 and Figure 4), suggesting that SNP49 may be a functional polymorphism that is in or near a locus involved in the control of α-cellulose content. This conjecture was also supported by the significant differences in expression among three genotype classes of SNP49 in either association or linkage populations (Figure 6A).

Fibers are the most abundant secondary wall–containing cells in wood of dicot species. During secondary wall formation, highly coordinated expression of multiple genes controls cell elongation or secondary wall thickening of fibers (Burton *et al.* 2004; Zhong *et al.* 2006). For example, *AtCesA7*/*IRX3* and *AtCOBL4*/*IRX6* are coexpressed in tissues during secondary cell wall development, and loss-of-function mutation of either of these genes causes diminished cellulose content and loss of mechanical strength of the plant body (Brown *et al.* 2005). A mutant allele of *AtCesA7* in fra5 (fragile fiber 5) causes a severe decrease in cellulose content and the thickness of fibers (Zhong *et al.* 2003). Cellulose is a biopolymer that provides a major contribution to secondary cell wall formation during cell expansion and elongation (Xie *et al.* 2011). These early studies laid the research foundation for elucidation of a significant nonsynonymous association (SNP59) in exon 6 of *PtoCesA4* with fiber width in *P. tomentosa* by using linkage-LD mapping, which demonstrated modes of gene action consistent with additive effects (Table 3, Table 4, and Table 5). Furthermore, a haplotype-based association with fiber width (SNP57–SNP59) suggests that this locus may be located close to causative polymorphisms. This is consistent with the finding that nonsynonymous mutations play special roles in assigning functions to specific domains or motifs of the CESA (Zhong *et al.* 2003). Further analyzing the protein structure encoded by *PtoCesA4*, we found that the nonsynonymous mutation of amino acid 245 (Ser to Tyr) is close to the two putative transmembrane domains (TMDs) at the N-terminus (217–238 and 250–267), which are involved in CESA protein–protein interactions (Joshi *et al.* 2004), suggesting that this nonsynonymous locus may affect the TMDs and also affect regulation of gene expression related to fiber width. CESAs are membrane-spanning proteins and small side chain residues often occur at the TMDs as a requirement of helix folding and structural stability. Zhang *et al.* (2009) identified a missense mutation (G858R) in the fifth TMD of the rice ortholog of *PtoCesA4* (*OsCesA4*); this mutation affects protein abundance in the plasma membrane and results in abnormal cell wall biosynthesis. Additionally, novel point mutations in the TMD have also been reported to affect cellulose synthesis in *Arabidopsis* (Chen *et al.* 2005).

Many functional analyses of SNPs have examined coding regions and splicing sites in candidate genes related to wood traits that can alter proteins and mRNA splicing. However, SNPs in noncoding regulatory regions can also influence important biological regulation (Thumma *et al.* 2009; Beaulieu *et al.* 2011). d’Alesio *et al.* (2005) detected that several SNPs are predicted to be related to genes by influencing the binding affinity of transcription factors in the promoter region. In this work, we detected a significant marker (SNP 18) at 273 bp (T/A) upstream of the transcriptional start site of the *PtoCesA4* promoter (Figure 1). Genotypic effect analysis of α-cellulose content in either association or linkage population showed that the trees heterozygous (AT) for this marker showed higher average α-cellulose content than the homozygous trees (Figure 4), indicating overdominance. Moreover, a common haplotype (SNP16–SNP18) associated with α-cellulose traits and SNP18 were also determined by using the multi-SNPs association model (Table 4 and Table S3). These results support that SNP18 might have a regulatory effect on *PtoCesA4* expression, or it could to be in very strong LD with a nearby regulatory polymorphism; the detailed regulatory mechanisms of this locus will require further investigation. Although the mutation in the 5′ flanking region did not result in an amino acid changes, phenotypic traits can be affected because 5′UTRs play crucial roles in the regulation of gene expression, especially for transcriptional mRNA stability, translational efficiency, or subcellular localization (Miyamoto *et al.* 2007; Lin and Li 2012). In our study, SNP41, located in the 5′UTR of *PtoCesA4*, had a significant association (*Q* < 0.10) with α-cellulose in the association populations (Table 3). The estimated allelic effects of SNP41 on α-cellulose corresponded well with estimates of transcript levels (Figure 6B), *i.e.*, heterozygotes (CT) had both higher average α-cellulose content and higher transcript levels than the homozygotes (CC and TT), suggesting that SNP41 may be a functional polymorphism affecting the regulation of gene expression. However, our follow-up study of this SNP in the linkage population did not support this observation. Similarly, no replication case has been reported in several previous association studies of wood traits (Dillon *et al.* 2010, 2012; Du *et al.* 2013). The differences between association and linkage populations may explain the “lack of validation” for this association, including their genetic background, complex gene–environment interactions, mapping resolution, population structure, and age-dependent effects (Du *et al.* 2013). In this study, the linkage population has a limited genomic data set from both the parents and the interspecific genetic background of the female parent. Therefore, improved power to detect and validate associations in future experiments could be achieved by establishing validation populations with families, or clonal material, from the discovery population of the same species (Dillon *et al.* 2010).

### Conclusions

Tissue-specific expression profiles revealed that *PtoCesA4* is highly expressed during secondary cell wall formation. Therefore, selection of optimal candidate genes through different approaches, such as EST database searches, transcript abundance profiles, QTL mapping, and comparison of orthologs in a model or related species, is very important for identifying useful alleles located within functional genes controlling traits of interest (Neale and Savolainen 2004; Thumma *et al.* 2005). Our work revealed that the greater length may lead to slightly higher LD than candidate genes analyzed previously in *Populus* and combined with a recent genome-wide study of LD in *P. trichocarpa* (Slavov *et al.* 2012), suggesting that LD studies in *P. tomentosa* should focus on a better understanding of the variability of haplotype structure across the entire genome. Comparatively, the preliminary application of multi-SNP analysis in *PtoCesA4* suggests that it will be promising to conduct association studies with virtually all the related genes that share biological pathways and to have a more complete understanding of the genetic architecture of quantitative variation (Eckert *et al.* 2012; Resende *et al.* 2012). Wood quality traits are quantitative traits controlled by multiple genes, with a moderate to high degree of heritability; however, growth traits have relatively low heritability compared with wood property traits (Thumma *et al.*, 2010). Therefore, several significant SNP associations with wood traits, obtained using linkage-LD mapping approaches combined with RNA transcript abundance among the genotypes of each significant SNP, represent important progress toward the identification of allelic variation responsible for wood traits and the development of successful marker-aided selection in trees. In the coming years, the rapid development of high-throughput sequencing is very likely to drive association studies toward genome-wide studies in trees.

## Supplementary Material

Supporting Information
