# Supplementary material for: Allelic Variation in a Cellulose Synthase Gene (PtoCesA4) Associated with Growth and Wood Properties in Populus tomentosa
Source: G3 (Bethesda). 2013 Nov 1;3(11):2069–84. doi: 10.1534/g3.113.007724 (PMC3815066; doi:10.1534/g3.113.007724)
Supplement: Supporting Information [file supp_3_11_2069__index.html]

Allelic Variation in a Cellulose Synthase Gene (PtoCesA4) Associated with Growth and Wood Properties in Populus tomentosa — Supporting Information 

# Allelic Variation in a Cellulose Synthase Gene (*PtoCesA4*) Associated with Growth and Wood Properties in *Populus tomentosa*

## Supporting Information for Du *et al.*, 2013

**Files in this Data Supplement:**

- Supporting Information - Figures S1-S2, Tables S1-S4, and Files S1-S5 (PDF, 1 MB)
- Figure S1 - The phylogenetic tree of PtoCesA4 with CesAs of the other plants. (PDF, 484 KB)
- Figure S2 - Pairwise linkage disequilibrium (LD) (*r*2) between 92 common single nucleotide polymorphisms (SNP) markers (minor allele frequencies >0.10) located in the cellulose synthase gene (*PtoCesA4*) in *Populus tomentosa*. (PDF, 379 KB)
- Table S1 - Summary of all SNP markers from *PtoCesA4* used in both association and linkage analysis in this study. (PDF, 523 KB)
- Table S2 - Summary of significant SNP marker-trait pairs identified at the threshold of P less than or equal to 0.05, using the mixed linear model (MLM) in the *Populus tomentosa* association population. (PDF, 488 KB)
- Table S3 - A summary of genetic associations between SNPs and phenotypic traits detected in *Populus tomentosa* association population using a multilocus Bayesian model (BAMD). (PDF, 452 KB)
- Table S4 - Summary of significant SNP marker-trait pairs identified in *PtoCesA4* at the threshold of *P* less than or equal to 0.05, using the *Populus tomentosa* linkage population, "YX01" (*Populus. alba* x *P. glandulosa*) as the female and clone "LM 50" (*P. tomentosa*) as the male. (PDF, 479 KB)
- File S1 - The real-time PCR data for expression analysis of *PtoCesA4* in the *Populus tomentosa* different tissues with triplicate technical and triplicate biological repetitions, respectively. (.xlsx, 9 KB)
- File S2 - The phenotype data used in the SNP-traits association analysis in *Populus tomentosa* natural population. (.xlsx, 47 KB)
- File S3 - The genotype data used in the SNP-traits association analysis in *Populus tomentosa* natural population. (.xlsx, 173 KB)
- File S4 - The phenotype data used in the SNP-traits association analysis in *Populus tomentosa* linkage population. (.xlsx, 119 KB)
- File S5 - The genotype data used in the SNP-traits association analysis in *Populus tomentosa* linkage population. (.xlsx, 247 KB)
