# Supplementary material for: Allelic Variation in a Cellulose Synthase Gene (PtoCesA4) Associated with Growth and Wood Properties in Populus tomentosa
Source: G3 (Bethesda). 2013 Nov 1;3(11):2069–84. doi: 10.1534/g3.113.007724 (PMC3815066; doi:10.1534/g3.113.007724)
Supplement: Supporting Information [file supp_g3.113.007724_007724SI.pdf]

G3 · Genes | Genomes | Genetics

Allelic Variation in a Cellulose Synthase Gene (*PtoCesA4*) Associated with Growth and Wood Properties in *Populus tomentosa*

Qingzhang Du,[*,§,1] Baohua Xu,[*,§,1] Wei Pan,[*,§] Chenrui Gong,[*,§] Qingshi Wang,[*,§] Jiaxing Tian,[*,§] Bailian Li[*,§,†] and Deqiang Zhang[*,§,2]

[*]National Engineering Laboratory for Tree Breeding, College of Biological Sciences and Technology, Beijing Forestry University, Beijing 100083, P. R. China

[§]Key Laboratory of Genetics and Breeding in Forest Trees and Ornamental Plants, Ministry of Education, College of Biological Sciences and Technology, Beijing Forestry University, Beijing 100083, P. R. China

[†]Department of Forestry, North Carolina State University, Raleigh, NC 27695-8203 USA

Sequence data from this article has been deposited in the GenBank Data Library under the accession Nos.KC762249-KC762292

DOI: 10.1534/g3.113.007724

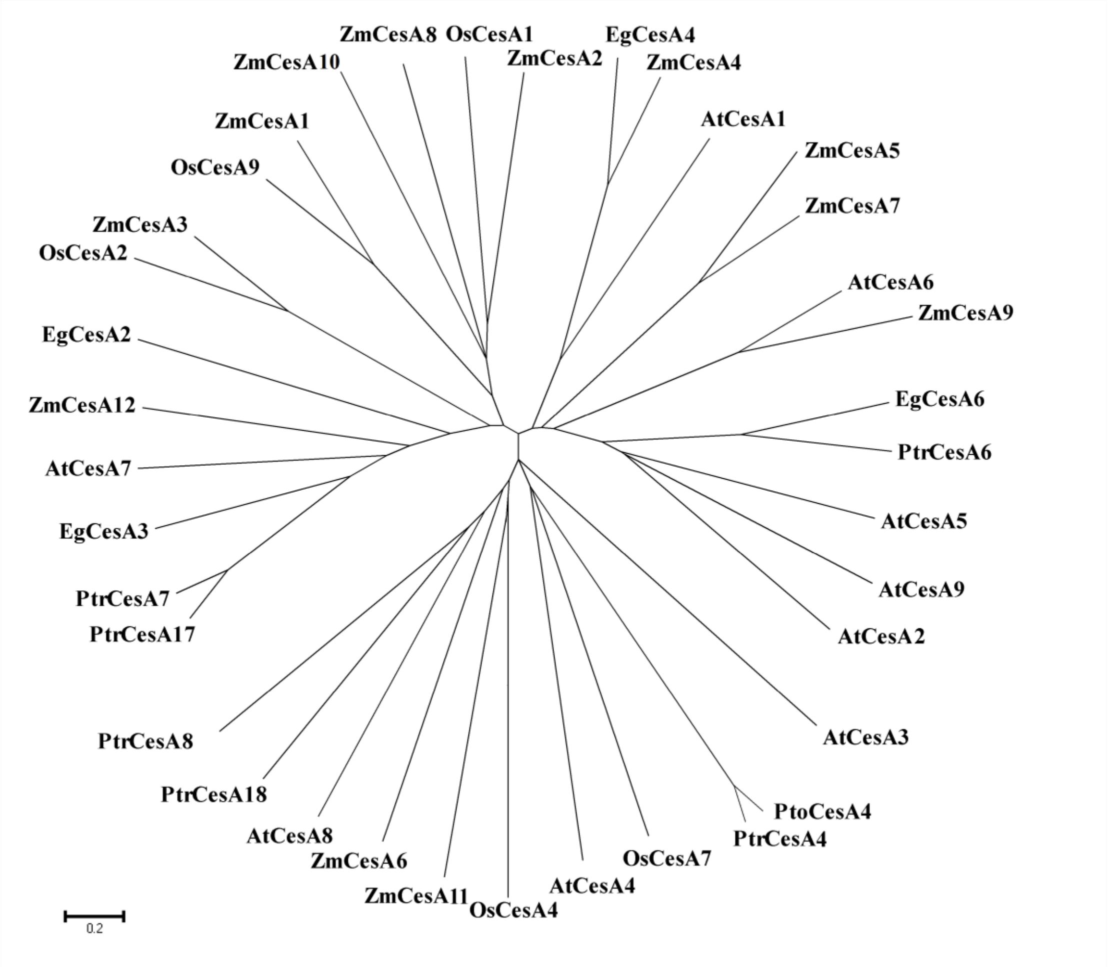

Figure S1 The phylogenetic tree of PtoCesA4 with CesAs of the other plants. Pto, *Populus tomentosa*; Ptr, *Populus trichocarpa*; At, *Arabidopsis thaliana*; Eg, *Eucalyptus grandis*; Os, *Oryza sativa*; Zm, *Zea mays*. Scale bar represents genetic distance.

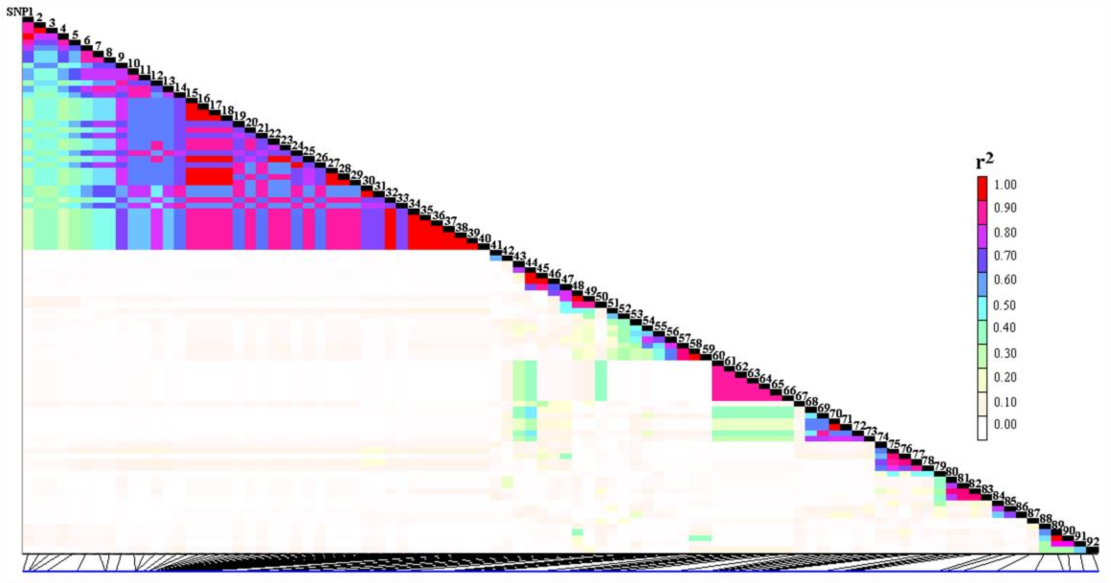

Figure S2 Pairwise linkage disequilibrium (LD) (r^2) between 92 common single nucleotide polymorphisms (SNP) markers (minor allele frequencies >0.10) located in the cellulose synthase gene (*PtoCesA4*) in *Populus tomentosa*. A larger number of SNPs were in linkage equilibrium ($r^2 < 0.3$, $P < 0.001$); limited LD of the SNP loci within the candidate gene did not extend over the entire gene region, and several LD blocks were identified, such as SNPs 15-18, SNPs 34-40, and SNPs 60-66 ($r^2 > 0.8$; $P < 0.001$).

Files S1-S5

Available for download at http://www.g3journal.org/lookup/suppl/doi:10.1534/g3.113.007724/-/DC1

File S1 The real-time PCR data for expression analysis of *PtoCesA4* in the *Populus tomentosa* different tissues with triplicate technical and triplicate biological repetitions, respectively.

File S2 The phenotype data used in the SNP-traits association analysis in *Populus tomentosa* natural population

File S3 The genotype data used in the SNP-traits association analysis in *Populus tomentosa* natural population

File S4 The phenotype data used in the SNP-traits association analysis in *Populus tomentosa* linkage population

File S5 The genotype data used in the SNP-traits association analysis in *Populus tomentosa* linkage population

Table S1 Summary of all SNP markers from *PtoCesA4* used in both association and linkage analysis in this study

SNPs	Position	Association population			Linkage population			
		**Code**	**Mutation**	**Minor allele (Frequency)**	**Code**	**Alleles of parents (Female : Male)**	**Expected segregation ratio**	***P*-value**
C-SNP-1	Promoter	SNP1	[T C]nc	C (0.31)	SNP1	[TC : TC]	1:2:1	NS
C-SNP-2	Promoter	SNP2	[G T]nc	G (0.37)	SNP2	[GT : GT]	1:2:1	NS
C-SNP-3	Promoter	SNP3	[A G]nc	A (0.11)	SNP3	[AA : AG]	1:1	NS
C-SNP-4	Promoter	SNP4	[T C]nc	C (0.32)				
C-SNP-5	Promoter	SNP5	[C A]nc	A (0.30)				
C-SNP-6	Promoter	SNP6	[G T]nc	T (0.33)	SNP6	[GG : TT]	/	/
C-SNP-7	Promoter	SNP7	[C G]nc	G (0.30)				
C-SNP-8	Promoter	SNP8	[G A]nc	A (0.30)	SNP8	[AA : AG]	1:1	NS
C-SNP-9	Promoter	SNP9	[C T]nc	C (0.32)				
C-SNP-10	Promoter	SNP10	[G A]nc	A (0.25)	SNP10	[GG : AG]	1:1	NS
C-SNP-11	Promoter	SNP11	[T C]nc	C (0.25)				
C-SNP-12	Promoter	SNP12	[A G]nc	A (0.35)				
C-SNP-13	Promoter	SNP13	[G A]nc	A (0.26)	SNP13	[GA : GA]	1:2:1	NS
C-SNP-14	Promoter	SNP14	[A G]nc	G (0.30)	SNP14	[TT : AA]	/	/
C-SNP-15	Promoter	SNP15	[G T]nc	G (0.30)	SNP15	[GT : GT]	1:2:1	NS
C-SNP-16	Promoter	SNP16	[C T]nc	C (0.32)				
C-SNP-17	Promoter	SNP17	[G A]nc	G (0.30)				
C-SNP-18	Promoter	SNP18	[T A]nc	T (0.28)	SNP18	[TT : AT]	1:1	NS
C-SNP-19	Promoter	SNP19	[C T]nc	T (0.30)				
C-SNP-20	Promoter	SNP20	[T C]nc	T (0.33)				
C-SNP-21	Promoter	SNP21	[G A]nc	A (0.30)	SNP21	[GA : GA]	1:2:1	NS
C-SNP-22	Promoter	SNP22	[G A]nc	G(0.32)	SNP22	[GG : GA]	1:1	NS

C-SNP-23	Promoter	SNP23	[T C][nc]	T (0.36)	SNP23	[TT : CC]	/	/
C-SNP-24	Promoter	SNP24	[A T][nc]	T (0.30)				
C-SNP-25	Promoter	SNP25	[G A][nc]	G (0.38)	SNP25	[GA : GA]	1:2:1	$P < 0.01$
C-SNP-26	Promoter	SNP26	[G A][nc]	A (0.30)				
C-SNP-27	Promoter	SNP27	[G T][nc]	G (0.38)				
C-SNP-28	Promoter	SNP28	[T A][nc]	T (0.37)	SNP28	[TA : TA]	1:2:1	NS
C-SNP-29	Promoter	SNP29	[C T][nc]	T (0.37)	SNP29	[CC : TT]	/	/
C-SNP-30	Promoter	SNP30	[G A][nc]	A (0.33)	SNP30	[GA : GA]	1:2:1	NS
C-SNP-31	Promoter	SNP31	[C T][nc]	T (0.32)	SNP31	[CT : TT]	1:1	NS
C-SNP-32	Promoter	SNP32	[A C][nc]	A (0.40)				
C-SNP-33	Promoter	SNP33	[T C][nc]	C (0.32)	SNP33	[CT : CT]	1:2:1	NS
C-SNP-34	Promoter	SNP34	[G T][nc]	G (0.40)				
C-SNP-35	Promoter	SNP35	[C T][nc]	C (0.40)				
C-SNP-36	Promoter	SNP36	[A T][nc]	A (0.40)	SNP36	[AT : AT]	1:2:1	NS
C-SNP-37	Promoter	SNP37	[G T][nc]	G (0.40)				
C-SNP-38	Promoter	SNP38	[T G][nc]	T (0.33)				
C-SNP-39	Promoter	SNP39	[G T][nc]	G (0.40)	SNP39	[GT : GG]	1:1	NS
C-SNP-40	Promoter	SNP40	[A G][nc]	A (0.40)				
C-SNP-41	5'UTR	SNP41	[T C][nc]	T (0.23)	SNP41	[CT : CT]	1:2:1	NS
C-SNP-42	5'UTR	SNP42	[C T][nc]	C (0.45)	SNP42	[CT : CT]	1:2:1	NS
C-SNP-43	Intron 1	SNP43	[T C][nc]	T (0.13)				
C-SNP-44	Intron 1	SNP44	[G T][nc]	T (0.15)	SNP44	[GT : GT]	1:2:1	NS
C-SNP-45	Exon 2	SNP45	[A C][s]	A (0.16)	SNP45	[AC : AA]	1:1	NS
C-SNP-46	Exon 2	SNP46	[T C][s]	T (0.40)	SNP46	[CT : CT]	1:2:1	NS
C-SNP-47	Exon 2	SNP47	[T C][s]	T (0.37)	SNP47	[TT : CT]	1:1	NS
C-SNP-48	Intron 2	SNP48	[A T][nc]	T (0.44)	SNP48	[AT : AA]	1:1	NS
C-SNP-49	Exon 3	SNP49	[C A][ns]	A (0.42)	SNP49	[AC : AC]	1:2:1	NS
C-SNP-50	Intron 3	SNP50	[C A][nc]	G (0.10)				

C-SNP-51	Intron 3	SNP51	[C A][nc]	A (0.32)	SNP51	[AC : AC]	1:2:1	NS
C-SNP-52	Intron 3	SNP52	[G T][nc]	T (0.47)				
C-SNP-53	Exon 4	SNP53	[C T][s]	C (0.35)				
C-SNP-54	Exon 5	SNP54	[A G][s]	A (0.42)	SNP54	[GG : AA]	/	/
C-SNP-55	Intron 5	SNP55	[T C][nc]	T (0.35)				
C-SNP-56	Intron 5	SNP56	[G A][nc]	G (0.43)	SNP56	[AG : AG]	1:2:1	NS
C-SNP-57	Exon 6	SNP57	[T C][s]	T (0.38)	SNP57	[TT : TC]	1:1	NS
C-SNP-58	Exon 6	SNP58	[T C][s]	T (0.40)				
C-SNP-59	Exon 6	SNP59	[A C][ns]	A (0.38)	SNP59	[AA : AC]	1:1	NS
C-SNP-60	Exon 6	SNP60	[G C][s]	C (0.13)				
C-SNP-61	Exon 6	SNP61	[T C][s]	C (0.13)				
C-SNP-62	Intron 6	SNP62	[T G][nc]	G (0.13)	SNP62	[TT : TG]	1:1	NS
C-SNP-63	Intron 6	SNP63	[T C][nc]	C (0.13)				
C-SNP-64	Intron 6	SNP64	[T C][nc]	C (0.13)	SNP64	[TC : TC]	1:2:1	NS
C-SNP-65	Intron 6	SNP65	[G A][nc]	A (0.13)	SNP65	[AA : GG]	/	/
C-SNP-66	Exon 7	SNP66	[A G][s]	G (0.13)				
C-SNP-67	Exon 7	SNP67	[G C][s]	C (0.13)				
C-SNP-68	Exon 9	SNP68	[T G][s]	G (0.10)	SNP68	[TG : TG]	1:2:1	NS
C-SNP-69	Exon 9	SNP69	[T C][s]	C (0.10)				
C-SNP-70	Intron 9	SNP70	[A T][nc]	T (0.15)	SNP70	[AT : AT]	1:2:1	NS
C-SNP-71	Intron 9	SNP71	[C A][nc]	A (0.15)				
C-SNP-72	Intron 9	SNP72	[T A][nc]	A (0.10)	SNP72	[AT : TT]	1:1	NS
C-SNP-73	Exon 10	SNP73	[G A][s]	A (0.12)				
C-SNP-74	Exon 10	SNP74	[A G][s]	A (0.45)				
C-SNP-75	Exon 10	SNP75	[T C][s]	T (0.43)	SNP75	[CT : CT]	1:2:1	NS
C-SNP-76	Exon 10	SNP76	[T C][s]	C (0.47)				
C-SNP-77	Exon 10	SNP77	[A G][s]	A (0.42)	SNP77	[AG : AG]	1:2:1	$P < 0.01$
C-SNP-78	Exon 10	SNP78	[G A][s]	G (0.40)				

C-SNP-79	Intron 10	SNP79	[T A][nc]	A (0.33)	SNP79	[AT : AA]	1:1	NS
C-SNP-80	Intron 10	SNP80	[A G][nc]	A (0.33)				
C-SNP-81	Intron 10	SNP81	[T C][nc]	A (0.46)	SNP81	[TT : CT]	1:1	NS
C-SNP-82	Intron 10	SNP82	[A T][nc]	A (0.42)				
C-SNP-83	Intron 10	SNP83	[G A][nc]	G (0.27)				
C-SNP-84	Intron 11	SNP84	[G A][nc]	A (0.35)	SNP84	[AG : AG]	1:2:1	NS
C-SNP-85	Intron 11	SNP85	[T G][nc]	G (0.35)	SNP85	[AA : AG]	1:1	$P < 0.01$
C-SNP-86	Exon 12	SNP86	[T C][s]	T (0.42)	SNP86	[TC : TC]	1:2:1	NS
C-SNP-87	Exon 12	SNP87	[T C][s]	T (0.42)				
C-SNP-88	Exon 12	SNP88	[G A][ns]	A (0.37)	SNP88	[AG : AG]	1:2:1	NS
C-SNP-89	3'UTR	SNP89	[G C][nc]	C (0.32)	SNP89	[CG : CG]	1:2:1	NS
C-SNP-90	3'UTR	SNP90	[G A][nc]	A (0.35)				
C-SNP-91	3'UTR	SNP91	[T A][nc]	A (0.37)	SNP91	[AT : AA]	1:1	NS
C-SNP-92	3'UTR	SNP92	[T C][nc]	C (0.38)				
NSNP-1	Promoter				NSNP1	[CC : TC]	1:1	NS
NSNP-2	Promoter				NSNP2	[AA : AT]	1:1	NS
NSNP-3	Promoter				NSNP3	[AG : AG]	1:2:1	NS
NSNP-4	Intron 3				NSNP4	[GG : GT]	1:1	$P < 0.01$
NSNP-5	Intron 10				NSNP5	[AG : AG]	1:2:1	NS
NSNP-6	3'UTR				NSNP6	[TT : CT]	1:1	NS

"C-SNP-" represents the common SNP identified in association mapping with the minor allele frequencies >0.10; "NSNP-" represents the novel unique SNPs identified in the parents of the linkage population; nonsynonymous polymorphism (ns); synonymous polymorphism (s); noncoding polymorphism (nc);. Not significant (NS); Not applied (/); the χ2 significance level of was $P < 0.01$.

Table S2 Summary of significant SNP marker-trait pairs identified at the threshold of *P* ≤ 0.05, using the mixed linear model (MLM) in the *Populus tomentosa* association population.

Trait	Locus	Position	Mutation	Association population （*N* = 460）		
				***P*-value**	***Q*-value**	***R*²(%)**
Lignin						
	SNP2	Promoter	[G : T]	0.0069	*Q* > 0.10	3.4
	SNP24	Promoter	[A : T]	0.0434	*Q* > 0.10	3.0
	SNP44	Intron1	[G : T]	0.0012	0.0551	2.1
	SNP47	Exon 2	[T : C]	0.0500	*Q* > 0.10	1.9
	SNP49	Exon 3	[C : A]	0.0025	0.0810	3.0
α-cellulose						
	SNP3	Promoter	[A : G]	0.0015	0.0629	2.2
	SNP18	Promoter	[T : A]	0.0011	0.0551	2.5
	SNP36	promoter	[A : T]	0.0061	*Q* > 0.10	3.5
	SNP41	5’UTR	[T : C]	3.02E-05	0.0035	1.6
	SNP48	Intron 2	[A : T]	0.0400	*Q* > 0.10	2.2
	SNP49	Exon 3	[C : A]	0.0031	0.0948	5.3
	SNP50	Intron 3	[C : A]	0.0142	*Q* > 0.10	1.7
Holocellulose						
	SNP27	Promoter	[G : T]	0.0281	*Q* > 0.10	6.0
	SNP38	Promoter	[T : G]	0.0052	*Q* > 0.10	2.9
	SNP45	Exon 2	[A : C]	0.0002	0.0142	4.0
	SNP48	Intron 2	[A : T]	0.0351	*Q* > 0.10	4.4
	SNP62	Intron 6	[T : G]	0.0104	*Q* > 0.10	3.4
	SNP70	Intron 9	[A : T]	0.0069	*Q* > 0.10	2.5
	SNP81	Intron 10	[T : C]	0.0002	0.0142	3.0
	SNP88	Exon 12	[G : A]	0.0161	*Q* > 0.10	1.9
Fiber length						
	SNP2	Promoter	[G : T]	0.0355	*Q* > 0.10	6.7
	SNP44	Intron1	[G : T]	0.0194	*Q* > 0.10	2.0
	SNP89	3’UTR	[G : C]	0.0070	*Q* > 0.10	1.5
	SNP90	3’UTR	[G : A]	0.0122	*Q* > 0.10	1.9
Fiber width						
	SNP58	Exon 6	[T: C]	0.0500	*Q* > 0.10	3.9
	SNP59	Exon 6	[A : C]	0.0008	0.0440	2.6
	SNP62	Intron 6	[T : G]	0.0475	*Q* > 0.10	1.8
	SNP70	Intron 9	[A : T]	0.0477	*Q* > 0.10	1.8
Microfibril angle (MFA)						
	SNP10	Promoter	[G : A]	0.0372	*Q* > 0.10	1.7
	SNP47	Exon 2	[T : C]	0.0195	*Q* > 0.10	2.6

Diameter at breast height (D)						
	SNP48	Intron 2	[A : T]	0.0009	0.0454	1.9
	SNP59	Exon 6	[A : C]	0.0151	*Q* > 0.10	3.0
	SNP62	Intron 6	[T : G]	0.0195	*Q* > 0.10	2.0
	SNP75	Exon 10	[T : C]	3.15E-05	0.0035	3.2
	SNP81	Intron 10	[T : C]	0.0003	0.0195	2.0
Tree height (H)						
	SNP44	Intron1	[G : T]	0.0179	*Q* > 0.10	1.2
	SNP49	Exon 3	[A : T]	0.0012	0.0551	4.3
	SNP89	3'UTR	[G : C]	0.0351	*Q* > 0.10	2.1
Stem volume(V)						
	SNP75	Exon 10	[T : C]	3.02E-05	0.0035	4.6
	SNP81	Intron 10	[T : C]	0.0143	*Q* > 0.10	2.0

R^2 = percentage of the phenotypic variance explained. *Q*-value = correction for multiple testing [false discovery rate FDR (*Q*) ≤ 0.10]

Table S3 A summary of genetic associations between SNPs and phenotypic traits detected in *Populus tomentosa* association population using a multilocus Bayesian model (BAMD)

Trait	Number of SNPs	R^2	Locus	Position	Mutation
Lignin	4	0.102			
			SNP24	Promoter	$[A : T]^{nc}$
			SNP44	Intron1	$[G : T]^{nc}$
			SNP49	Exon 3	$[C : A]^{ns}$
			SNP60	Exon 6	$[G : C]^{s}$
α-cellulose	7	0.124			
			SNP3	Promoter	$[A : G]^{nc}$
			SNP18	Promoter	$[T : A]^{nc}$
			SNP36	promoter	$[A : T]^{nc}$
			SNP41	5'UTR	$[T : C]^{nc}$
			SNP49	Exon 3	$[C : A]^{ns}$
			SNP62	Intron 6	$[T : G]^{nc}$
			SNP81	Intron 10	$[T : C]^{nc}$
Holocellulose	5	0.090			
			SNP45	Exon 2	$[A : C]^{s}$
			SNP48	Intron 2	$[A : T]^{nc}$
			SNP66	Exon 7	$[A : G]^{s}$
			SNP81	Intron 10	$[T : C]^{nc}$
			SNP88	Exon 12	$[G : A]^{ns}$
Fiber length	3	0.066			
			SNP44	Intron1	$[G : T]^{nc}$
			SNP75	Exon 10	$[T : C]^{s}$
			SNP89	3'UTR	$[G : C]^{nc}$
Fiber width	4	0.106			
			SNP23	promoter	$[T : C]^{nc}$
			SNP59	Exon 6	$[A : C]^{ns}$
			SNP70	Intron 9	$[A : T]^{nc}$
			SNP90	3'UTR	$[G : A]^{nc}$
Microfibril angle (MFA)	3	0.071			
			SNP7	promoter	$[C : G]^{nc}$
			SNP18	Promoter	$[T : A]^{nc}$
			SNP48	Intron 2	$[A : T]^{nc}$
Diameter at breast height (D)	7	0.090			
			SNP23	promoter	$[T : C]^{nc}$
			SNP36	promoter	$[A : T]^{nc}$
			SNP48	Intron 2	$[A : T]^{nc}$

			SNP62	Intron 6	[T : G]^{nc}
			SNP75	Exon 10	[T : C]^{s}
			SNP80	Intron 10	[A : G]^{nc}
			SNP81	Intron 10	[T : C]^{nc}
Tree height (H)	2	0.039			
			SNP49	Exon 3	[A : T]^{ns}
			SNP81	Intron 10	[T : C]^{nc}
Stem volume(V)	3	0.067			
			SNP36	promoter	[A : T]^{nc}
			SNP75	Exon 10	[T : C]^{s}
			SNP80	Intron 10	[A : G]^{nc}

R^2 = percentage of the phenotypic variance explained; nonsynonymous polymorphism (ns); synonymous polymorphism (s); noncoding polymorphism (nc).

Table S4 Summary of significant SNP marker-trait pairs identified in *PtoCesA4* at the threshold of $P \leq 0.05$, using the *Populus tomentosa* linkage population, "YX01" (*Populus. alba* × *P. glandulosa*) as the female and clone "LM 50" (*P. tomentosa*) as the male.

Trait	Locus	Position	Alleles of parents (Female : Male)	Linkage population (*N* = 1200)		
				P-value	*Q*-value	R^2 (%)
Lignin						
	SNP3	Promoter	[AA : AG]	0.0070	*Q* > 0.10	1.3
	SNP49	Exon 3	[AC : AC]	0.0105	*Q* > 0.10	2.0
α-cellulose						
	SNP18	Promoter	[TT : AT]	0.0036	0.0693	2.8
	SNP49	Exon 3	[AC : AC]	0.0015	0.0490	3.6
	SNP75	Exon 10	[CT : CT]	0.0019	0.0532	1.5
	SNP91	3'UTR	[AT : AA]	0.0104	*Q* > 0.10	2.3
Holocellulose						
	SNP18	Promoter	[AT : AT]	0.0281	*Q* > 0.10	3.0
	SNP45	Exon 2	[AC : AA]	0.0344	*Q* > 0.10	3.4
	SNP88	Exon 12	[AG : AG]	0.0034	0.0693	1.9
Fiber length						
	SNP59	Exon 6	[AA : AC]	0.0423	*Q* > 0.10	2.7
	SNP70	Intron 9	[AT : AT]	0.0013	0.0490	3.0
Fiber width						
	SNP44	Intron1	[GT : GT]	0.0082	*Q* > 0.10	1.9
	SNP59	Exon 6	[AA : AC]	0.0044	0.0693	2.5
Diameter at breast height (D)						
	SNP89	3'UTR	[CG : CG]	0.0100	*Q* > 0.10	4.0
Tree height (H)						
	SNP51	Intron3	[AC : AC]	2.55E-05	0.0050	3.0
	SNP89	3'UTR	[CG : CG]	0.0351	*Q* > 0.10	2.1
Stem volume(V)						

	SNP18	Promoter	[AT : AT]	0.0093	$Q > 0.10$	1.3
	SNP75	Exon 10	[CT : CT]	0.0088	$Q > 0.10$	5.4

R^2 = percentage of the phenotypic variance explained. *Q*-value = correction for multiple testing [false discovery rate FDR (Q) ≤ 0.10]
