# Supplementary material for: Allelic Variation in a Cellulose Synthase Gene (PtoCesA4) Associated with Growth and Wood Properties in Populus tomentosa
Source: G3 (Bethesda). 2013 Nov 1;3(11):2069–84. doi: 10.1534/g3.113.007724 (PMC3815066; doi:10.1534/g3.113.007724)
Supplement: Supporting Information [file supp_g3.113.007724_FigureS1.pdf]

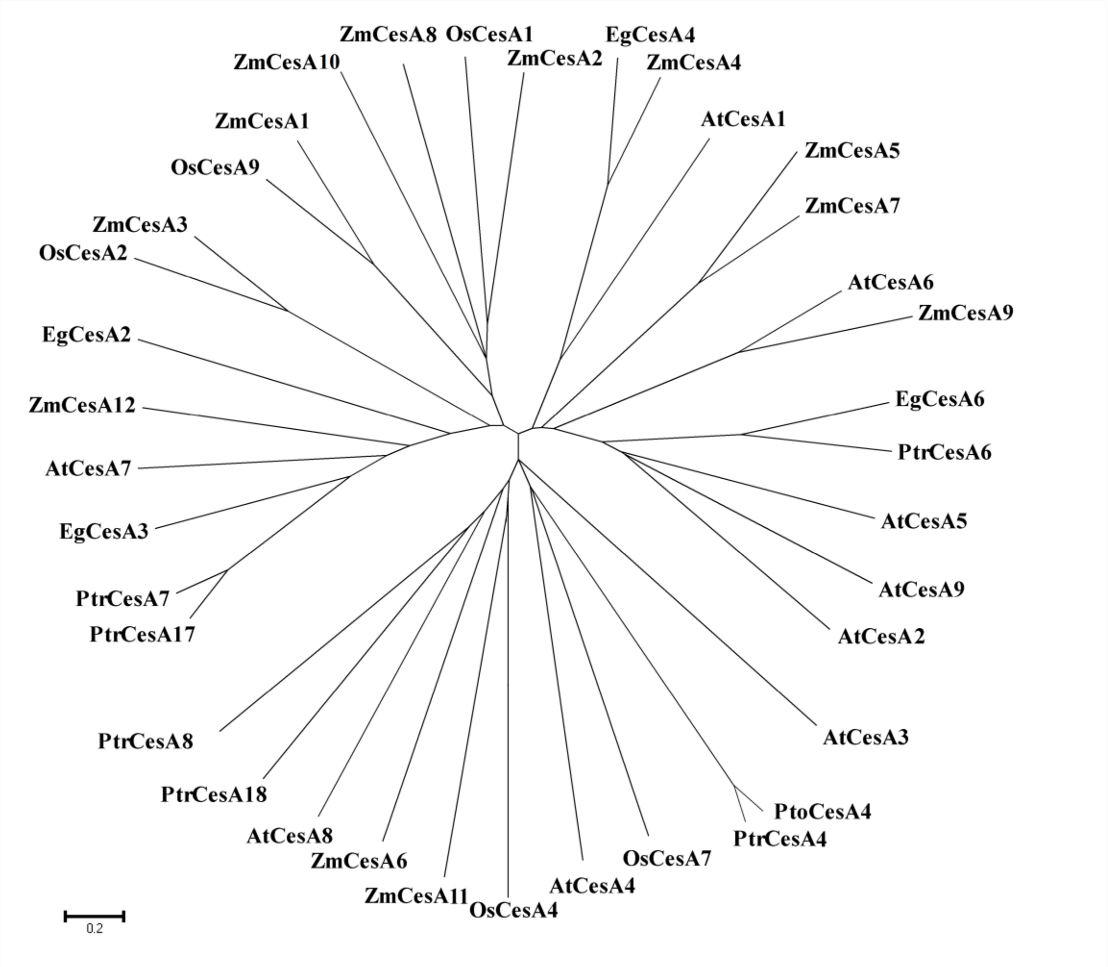

Figure S1 The phylogenetic tree of PtoCesA4 with CesAs of the other plants. Pto, *Populus tomentosa*; Ptr, *Populus trichocarpa*; At, *Arabidopsis thaliana*; Eg, *Eucalyptus grandis*; Os, *Oryza sativa*; Zm, *Zea mays*. Scale bar represents genetic distance.
