# Supplementary material for: Allelic Variation in a Cellulose Synthase Gene (PtoCesA4) Associated with Growth and Wood Properties in Populus tomentosa
Source: G3 (Bethesda). 2013 Nov 1;3(11):2069–84. doi: 10.1534/g3.113.007724 (PMC3815066; doi:10.1534/g3.113.007724)
Supplement: Supporting Information [file supp_g3.113.007724_FigureS2.pdf]

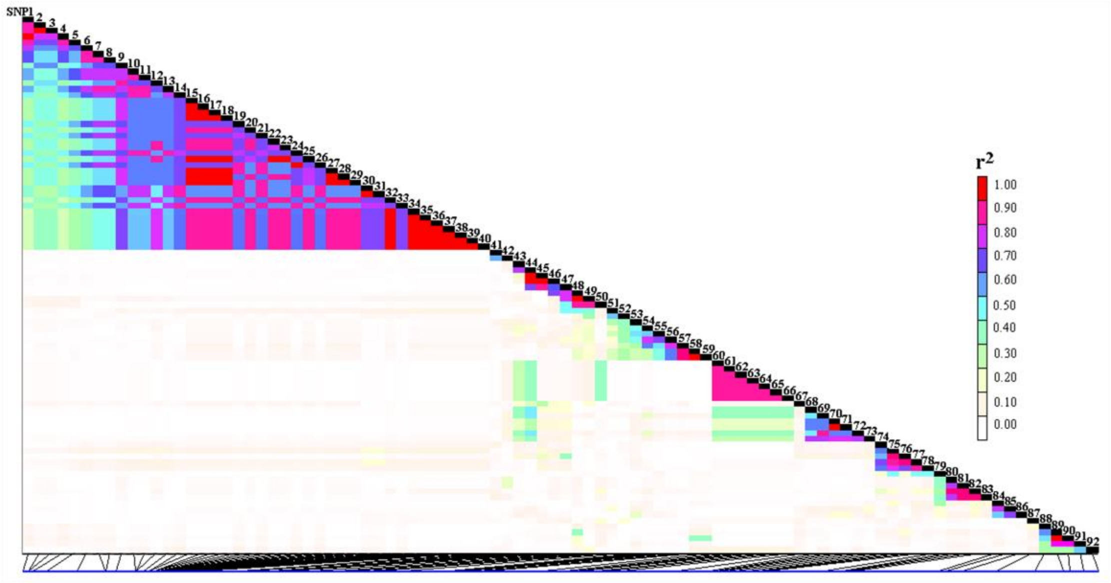

Figure S2 Pairwise linkage disequilibrium (LD) (r^2) between 92 common single nucleotide polymorphisms (SNP) markers (minor allele frequencies >0.10) located in the cellulose synthase gene (*PtoCesA4*) in *Populus tomentosa*. A larger number of SNPs were in linkage equilibrium ($r^2 < 0.3$, P < 0.001); limited LD of the SNP loci within the candidate gene did not extend over the entire gene region, and several LD blocks were identified, such as SNPs 15-18, SNPs 34-40, and SNPs 60-66 ($r^2 > 0.8$; P < 0.001).
