# Supplementary material for: Allelic Variation in a Cellulose Synthase Gene (PtoCesA4) Associated with Growth and Wood Properties in Populus tomentosa
Source: G3 (Bethesda). 2013 Nov 1;3(11):2069–84. doi: 10.1534/g3.113.007724 (PMC3815066; doi:10.1534/g3.113.007724)
Supplement: Supporting Information [file supp_g3.113.007724_TableS1.pdf]

Table S1 Summary of all SNP markers from *PtoCesA4* used in both association and linkage analysis in this study

**SNPs**	**Position**	**Association population**			**Linkage population**			
		**Code**	**Mutation**	**Minor allele** (Frequency)	**Code**	**Alleles of parents** (Female : Male)	**Expected segregation ratio**	***P*-value**
C-SNP-1	Promoter	SNP1	[T C]^{nc}	C (0.31)	SNP1	[TC : TC]	1:2:1	NS
C-SNP-2	Promoter	SNP2	[G T]^{nc}	G (0.37)	SNP2	[GT : GT]	1:2:1	NS
C-SNP-3	Promoter	SNP3	[A G]^{nc}	A (0.11)	SNP3	[AA : AG]	1:1	NS
C-SNP-4	Promoter	SNP4	[T C]^{nc}	C (0.32)				
C-SNP-5	Promoter	SNP5	[C A]^{nc}	A (0.30)				
C-SNP-6	Promoter	SNP6	[G T]^{nc}	T (0.33)	SNP6	[GG : TT]	/	/
C-SNP-7	Promoter	SNP7	[C G]^{nc}	G (0.30)				
C-SNP-8	Promoter	SNP8	[G A]^{nc}	A (0.30)	SNP8	[AA : AG]	1:1	NS
C-SNP-9	Promoter	SNP9	[C T]^{nc}	C (0.32)				
C-SNP-10	Promoter	SNP10	[G A]^{nc}	A (0.25)	SNP10	[GG : AG]	1:1	NS
C-SNP-11	Promoter	SNP11	[T C]^{nc}	C (0.25)				
C-SNP-12	Promoter	SNP12	[A G]^{nc}	A (0.35)				
C-SNP-13	Promoter	SNP13	[G A]^{nc}	A (0.26)	SNP13	[GA : GA]	1:2:1	NS
C-SNP-14	Promoter	SNP14	[A G]^{nc}	G (0.30)	SNP14	[TT : AA]	/	/
C-SNP-15	Promoter	SNP15	[G T]^{nc}	G (0.30)	SNP15	[GT : GT]	1:2:1	NS
C-SNP-16	Promoter	SNP16	[C T]^{nc}	C (0.32)				
C-SNP-17	Promoter	SNP17	[G A]^{nc}	G (0.30)				
C-SNP-18	Promoter	SNP18	[T A]^{nc}	T (0.28)	SNP18	[TT : AT]	1:1	NS
C-SNP-19	Promoter	SNP19	[C T]^{nc}	T (0.30)				
C-SNP-20	Promoter	SNP20	[T C]^{nc}	T (0.33)				
C-SNP-21	Promoter	SNP21	[G A]^{nc}	A (0.30)	SNP21	[GA : GA]	1:2:1	NS
C-SNP-22	Promoter	SNP22	[G A]^{nc}	G(0.32)	SNP22	[GG : GA]	1:1	NS

C-SNP-23	Promoter	SNP23	[T C]nc	T (0.36)	SNP23	[TT : CC]	/	/
C-SNP-24	Promoter	SNP24	[A T]nc	T (0.30)				
C-SNP-25	Promoter	SNP25	[G A]nc	G (0.38)	SNP25	[GA : GA]	1:2:1	*P* < 0.01
C-SNP-26	Promoter	SNP26	[G A]nc	A (0.30)				
C-SNP-27	Promoter	SNP27	[G T]nc	G (0.38)				
C-SNP-28	Promoter	SNP28	[T A]nc	T (0.37)	SNP28	[TA : TA]	1:2:1	NS
C-SNP-29	Promoter	SNP29	[C T]nc	T (0.37)	SNP29	[CC : TT]	/	/
C-SNP-30	Promoter	SNP30	[G A]nc	A (0.33)	SNP30	[GA : GA]	1:2:1	NS
C-SNP-31	Promoter	SNP31	[C T]nc	T (0.32)	SNP31	[CT : TT]	1:1	NS
C-SNP-32	Promoter	SNP32	[A C]nc	A (0.40)				
C-SNP-33	Promoter	SNP33	[T C]nc	C (0.32)	SNP33	[CT : CT]	1:2:1	NS
C-SNP-34	Promoter	SNP34	[G T]nc	G (0.40)				
C-SNP-35	Promoter	SNP35	[C T]nc	C (0.40)				
C-SNP-36	Promoter	SNP36	[A T]nc	A (0.40)	SNP36	[AT : AT]	1:2:1	NS
C-SNP-37	Promoter	SNP37	[G T]nc	G (0.40)				
C-SNP-38	Promoter	SNP38	[T G]nc	T (0.33)				
C-SNP-39	Promoter	SNP39	[G T]nc	G (0.40)	SNP39	[GT : GG]	1:1	NS
C-SNP-40	Promoter	SNP40	[A G]nc	A (0.40)				
C-SNP-41	5'UTR	SNP41	[T C]nc	T (0.23)	SNP41	[CT : CT]	1:2:1	NS
C-SNP-42	5'UTR	SNP42	[C T]nc	C (0.45)	SNP42	[CT : CT]	1:2:1	NS
C-SNP-43	Intron 1	SNP43	[T C]nc	T (0.13)				
C-SNP-44	Intron 1	SNP44	[G T]nc	T (0.15)	SNP44	[GT : GT]	1:2:1	NS
C-SNP-45	Exon 2	SNP45	[A C]s	A (0.16)	SNP45	[AC : AA]	1:1	NS
C-SNP-46	Exon 2	SNP46	[T C]s	T (0.40)	SNP46	[CT : CT]	1:2:1	NS
C-SNP-47	Exon 2	SNP47	[T C]s	T (0.37)	SNP47	[TT : CT]	1:1	NS
C-SNP-48	Intron 2	SNP48	[A T]nc	T (0.44)	SNP48	[AT : AA]	1:1	NS
C-SNP-49	Exon 3	SNP49	[C A]ns	A (0.42)	SNP49	[AC : AC]	1:2:1	NS
C-SNP-50	Intron 3	SNP50	[C A]nc	G (0.10)				

C-SNP-51	Intron 3	SNP51	[C A]^{nc}	A (0.32)	SNP51	[AC : AC]	1:2:1	NS
C-SNP-52	Intron 3	SNP52	[G T]^{nc}	T (0.47)				
C-SNP-53	Exon 4	SNP53	[C T]^{s}	C (0.35)				
C-SNP-54	Exon 5	SNP54	[A G]^{s}	A (0.42)	SNP54	[GG : AA]	/	/
C-SNP-55	Intron 5	SNP55	[T C]^{nc}	T (0.35)				
C-SNP-56	Intron 5	SNP56	[G A]^{nc}	G (0.43)	SNP56	[AG : AG]	1:2:1	NS
C-SNP-57	Exon 6	SNP57	[T C]^{s}	T (0.38)	SNP57	[TT : TC]	1:1	NS
C-SNP-58	Exon 6	SNP58	[T C]^{s}	T (0.40)				
C-SNP-59	Exon 6	SNP59	[A C]^{ns}	A (0.38)	SNP59	[AA : AC]	1:1	NS
C-SNP-60	Exon 6	SNP60	[G C]^{s}	C (0.13)				
C-SNP-61	Exon 6	SNP61	[T C]^{s}	C (0.13)				
C-SNP-62	Intron 6	SNP62	[T G]^{nc}	G (0.13)	SNP62	[TT : TG]	1:1	NS
C-SNP-63	Intron 6	SNP63	[T C]^{nc}	C (0.13)				
C-SNP-64	Intron 6	SNP64	[T C]^{nc}	C (0.13)	SNP64	[TC : TC]	1:2:1	NS
C-SNP-65	Intron 6	SNP65	[G A]^{nc}	A (0.13)	SNP65	[AA : GG]	/	/
C-SNP-66	Exon 7	SNP66	[A G]^{s}	G (0.13)				
C-SNP-67	Exon 7	SNP67	[G C]^{s}	C (0.13)				
C-SNP-68	Exon 9	SNP68	[T G]^{s}	G (0.10)	SNP68	[TG : TG]	1:2:1	NS
C-SNP-69	Exon 9	SNP69	[T C]^{s}	C (0.10)				
C-SNP-70	Intron 9	SNP70	[A T]^{nc}	T (0.15)	SNP70	[AT : AT]	1:2:1	NS
C-SNP-71	Intron 9	SNP71	[C A]^{nc}	A (0.15)				
C-SNP-72	Intron 9	SNP72	[T A]^{nc}	A (0.10)	SNP72	[AT : TT]	1:1	NS
C-SNP-73	Exon 10	SNP73	[G A]^{s}	A (0.12)				
C-SNP-74	Exon 10	SNP74	[A G]^{s}	A (0.45)				
C-SNP-75	Exon 10	SNP75	[T C]^{s}	T (0.43)	SNP75	[CT : CT]	1:2:1	NS
C-SNP-76	Exon 10	SNP76	[T C]^{s}	C (0.47)				
C-SNP-77	Exon 10	SNP77	[A G]^{s}	A (0.42)	SNP77	[AG : AG]	1:2:1	$P < 0.01$
C-SNP-78	Exon 10	SNP78	[G A]^{s}	G (0.40)				

C-SNP-79	Intron 10	SNP79	[T A][nc]	A (0.33)	SNP79	[AT : AA]	1:1	NS
C-SNP-80	Intron 10	SNP80	[A G][nc]	A (0.33)				
C-SNP-81	Intron 10	SNP81	[T C][nc]	A (0.46)	SNP81	[TT : CT]	1:1	NS
C-SNP-82	Intron 10	SNP82	[A T][nc]	A (0.42)				
C-SNP-83	Intron 10	SNP83	[G A][nc]	G (0.27)				
C-SNP-84	Intron 11	SNP84	[G A][nc]	A (0.35)	SNP84	[AG : AG]	1:2:1	NS
C-SNP-85	Intron 11	SNP85	[T G][nc]	G (0.35)	SNP85	[AA : AG]	1:1	$P < 0.01$
C-SNP-86	Exon 12	SNP86	[T C][s]	T (0.42)	SNP86	[TC : TC]	1:2:1	NS
C-SNP-87	Exon 12	SNP87	[T C][s]	T (0.42)				
C-SNP-88	Exon 12	SNP88	[G A][ns]	A (0.37)	SNP88	[AG : AG]	1:2:1	NS
C-SNP-89	3'UTR	SNP89	[G C][nc]	C (0.32)	SNP89	[CG : CG]	1:2:1	NS
C-SNP-90	3'UTR	SNP90	[G A][nc]	A (0.35)				
C-SNP-91	3'UTR	SNP91	[T A][nc]	A (0.37)	SNP91	[AT : AA]	1:1	NS
C-SNP-92	3'UTR	SNP92	[T C][nc]	C (0.38)				
NSNP-1	Promoter				NSNP1	[CC : TC]	1:1	NS
NSNP-2	Promoter				NSNP2	[AA : AT]	1:1	NS
NSNP-3	Promoter				NSNP3	[AG : AG]	1:2:1	NS
NSNP-4	Intron 3				NSNP4	[GG : GT]	1:1	$P < 0.01$
NSNP-5	Intron 10				NSNP5	[AG : AG]	1:2:1	NS
NSNP-6	3'UTR				NSNP6	[TT : CT]	1:1	NS

"C-SNP-" represents the common SNP identified in association mapping with the minor allele frequencies >0.10; "NSNP-" represents the novel unique SNPs identified in the parents of the linkage population; nonsynonymous polymorphism (ns); synonymous polymorphism (s); noncoding polymorphism (nc);. Not significant (NS); Not applied (/); the $\chi 2$ significance level of was $P < 0.01$.
