# Supplementary material for: Allelic Variation in a Cellulose Synthase Gene (PtoCesA4) Associated with Growth and Wood Properties in Populus tomentosa
Source: G3 (Bethesda). 2013 Nov 1;3(11):2069–84. doi: 10.1534/g3.113.007724 (PMC3815066; doi:10.1534/g3.113.007724)
Supplement: Supporting Information [file supp_g3.113.007724_TableS2.pdf]

Table S2 Summary of significant SNP marker-trait pairs identified at the threshold of *P* ≤ 0.05, using the mixed linear model (MLM) in the *Populus tomentosa* association population.

Trait	Locus	Position	Mutation	Association population (*N* = 460)		
				***P*-value**	***Q*-value**	***R*²** (%)
Lignin						
	SNP2	Promoter	[G : T]	0.0069	*Q* > 0.10	3.4
	SNP24	Promoter	[A : T]	0.0434	*Q* > 0.10	3.0
	SNP44	Intron1	[G : T]	0.0012	0.0551	2.1
	SNP47	Exon 2	[T : C]	0.0500	*Q* > 0.10	1.9
	SNP49	Exon 3	[C : A]	0.0025	0.0810	3.0
α-cellulose						
	SNP3	Promoter	[A : G]	0.0015	0.0629	2.2
	SNP18	Promoter	[T : A]	0.0011	0.0551	2.5
	SNP36	promoter	[A : T]	0.0061	*Q* > 0.10	3.5
	SNP41	5'UTR	[T : C]	3.02E-05	0.0035	1.6
	SNP48	Intron 2	[A : T]	0.0400	*Q* > 0.10	2.2
	SNP49	Exon 3	[C : A]	0.0031	0.0948	5.3
	SNP50	Intron 3	[C : A]	0.0142	*Q* > 0.10	1.7
Holocellulose						
	SNP27	Promoter	[G : T]	0.0281	*Q* > 0.10	6.0
	SNP38	Promoter	[T : G]	0.0052	*Q* > 0.10	2.9
	SNP45	Exon 2	[A : C]	0.0002	0.0142	4.0
	SNP48	Intron 2	[A : T]	0.0351	*Q* > 0.10	4.4
	SNP62	Intron 6	[T : G]	0.0104	*Q* > 0.10	3.4
	SNP70	Intron 9	[A : T]	0.0069	*Q* > 0.10	2.5
	SNP81	Intron 10	[T : C]	0.0002	0.0142	3.0
	SNP88	Exon 12	[G : A]	0.0161	*Q* > 0.10	1.9
Fiber length						
	SNP2	Promoter	[G : T]	0.0355	*Q* > 0.10	6.7
	SNP44	Intron1	[G : T]	0.0194	*Q* > 0.10	2.0
	SNP89	3'UTR	[G : C]	0.0070	*Q* > 0.10	1.5
	SNP90	3'UTR	[G : A]	0.0122	*Q* > 0.10	1.9
Fiber width						
	SNP58	Exon 6	[T: C]	0.0500	*Q* > 0.10	3.9
	SNP59	Exon 6	[A : C]	0.0008	0.0440	2.6
	SNP62	Intron 6	[T : G]	0.0475	*Q* > 0.10	1.8
	SNP70	Intron 9	[A : T]	0.0477	*Q* > 0.10	1.8
Microfibril angle (MFA)						
	SNP10	Promoter	[G : A]	0.0372	*Q* > 0.10	1.7
	SNP47	Exon 2	[T : C]	0.0195	*Q* > 0.10	2.6

Diameter at breast height (D)						
	SNP48	Intron 2	[A : T]	0.0009	0.0454	1.9
	SNP59	Exon 6	[A : C]	0.0151	*Q* > 0.10	3.0
	SNP62	Intron 6	[T : G]	0.0195	*Q* > 0.10	2.0
	SNP75	Exon 10	[T : C]	3.15E-05	0.0035	3.2
	SNP81	Intron 10	[T : C]	0.0003	0.0195	2.0
Tree height (H)						
	SNP44	Intron1	[G : T]	0.0179	*Q* > 0.10	1.2
	SNP49	Exon 3	[A : T]	0.0012	0.0551	4.3
	SNP89	3'UTR	[G : C]	0.0351	*Q* > 0.10	2.1
Stem volume(V)						
	SNP75	Exon 10	[T : C]	3.02E-05	0.0035	4.6
	SNP81	Intron 10	[T : C]	0.0143	*Q* > 0.10	2.0

R^2 = percentage of the phenotypic variance explained. *Q*-value = correction for multiple testing [false discovery rate FDR (*Q*) ≤ 0.10]
