# Supplementary material for: Allelic Variation in a Cellulose Synthase Gene (PtoCesA4) Associated with Growth and Wood Properties in Populus tomentosa
Source: G3 (Bethesda). 2013 Nov 1;3(11):2069–84. doi: 10.1534/g3.113.007724 (PMC3815066; doi:10.1534/g3.113.007724)
Supplement: Supporting Information [file supp_g3.113.007724_TableS3.pdf]

Table S3 A summary of genetic associations between SNPs and phenotypic traits detected in *Populus tomentosa* association population using a multilocus Bayesian model (BAMD)

Trait	Number of SNPs	R^2	Locus	Position	Mutation
Lignin	4	0.102			
			SNP24	Promoter	$[A : T]^{nc}$
			SNP44	Intron1	$[G : T]^{nc}$
			SNP49	Exon 3	$[C : A]^{ns}$
			SNP60	Exon 6	$[G : C]^{s}$
α-cellulose	7	0.124			
			SNP3	Promoter	$[A : G]^{nc}$
			SNP18	Promoter	$[T : A]^{nc}$
			SNP36	promoter	$[A : T]^{nc}$
			SNP41	5'UTR	$[T : C]^{nc}$
			SNP49	Exon 3	$[C : A]^{ns}$
			SNP62	Intron 6	$[T : G]^{nc}$
			SNP81	Intron 10	$[T : C]^{nc}$
Holocellulose	5	0.090			
			SNP45	Exon 2	$[A : C]^{s}$
			SNP48	Intron 2	$[A : T]^{nc}$
			SNP66	Exon 7	$[A : G]^{s}$
			SNP81	Intron 10	$[T : C]^{nc}$
			SNP88	Exon 12	$[G : A]^{ns}$
Fiber length	3	0.066			
			SNP44	Intron1	$[G : T]^{nc}$
			SNP75	Exon 10	$[T : C]^{s}$
			SNP89	3'UTR	$[G : C]^{nc}$
Fiber width	4	0.106			
			SNP23	promoter	$[T : C]^{nc}$
			SNP59	Exon 6	$[A : C]^{ns}$
			SNP70	Intron 9	$[A : T]^{nc}$
			SNP90	3'UTR	$[G : A]^{nc}$
Microfibril angle (MFA)	3	0.071			
			SNP7	promoter	$[C : G]^{nc}$
			SNP18	Promoter	$[T : A]^{nc}$
			SNP48	Intron 2	$[A : T]^{nc}$
Diameter at breast height (D)	7	0.090			
			SNP23	promoter	$[T : C]^{nc}$
			SNP36	promoter	$[A : T]^{nc}$
			SNP48	Intron 2	$[A : T]^{nc}$

			SNP62	Intron 6	[T : G]^{nc}
			SNP75	Exon 10	[T : C]^{s}
			SNP80	Intron 10	[A : G]^{nc}
			SNP81	Intron 10	[T : C]^{nc}
Tree height (H)	2	0.039			
			SNP49	Exon 3	[A : T]^{ns}
			SNP81	Intron 10	[T : C]^{nc}
Stem volume(V)	3	0.067			
			SNP36	promoter	[A : T]^{nc}
			SNP75	Exon 10	[T : C]^{s}
			SNP80	Intron 10	[A : G]^{nc}

R^2 = percentage of the phenotypic variance explained; nonsynonymous polymorphism (ns); synonymous polymorphism (s); noncoding polymorphism (nc).
