# Supplementary material for: Allelic Variation in a Cellulose Synthase Gene (PtoCesA4) Associated with Growth and Wood Properties in Populus tomentosa
Source: G3 (Bethesda). 2013 Nov 1;3(11):2069–84. doi: 10.1534/g3.113.007724 (PMC3815066; doi:10.1534/g3.113.007724)
Supplement: Supporting Information [file supp_g3.113.007724_TableS4.pdf]

Table S4 Summary of significant SNP marker-trait pairs identified in *PtoCesA4* at the threshold of *P* ≤ 0.05, using the *Populus tomentosa* linkage population, "YX01" (*Populus*. *alba* × *P. glandulosa*) as the female and clone "LM 50" (*P. tomentosa*) as the male.

Trait	Locus	Position	Alleles of parents (Female : Male)	Linkage population (*N* = 1200) *P*-value	*Q*-value	R^2 (%)
Lignin						
	SNP3	Promoter	[AA : AG]	0.0070	*Q* > 0.10	1.3
	SNP49	Exon 3	[AC : AC]	0.0105	*Q* > 0.10	2.0
α-cellulose						
	SNP18	Promoter	[TT : AT]	0.0036	0.0693	2.8
	SNP49	Exon 3	[AC : AC]	0.0015	0.0490	3.6
	SNP75	Exon 10	[CT : CT]	0.0019	0.0532	1.5
	SNP91	3'UTR	[AT : AA]	0.0104	*Q* > 0.10	2.3
Holocellulose						
	SNP18	Promoter	[AT : AT]	0.0281	*Q* > 0.10	3.0
	SNP45	Exon 2	[AC : AA]	0.0344	*Q* > 0.10	3.4
	SNP88	Exon 12	[AG : AG]	0.0034	0.0693	1.9
Fiber length						
	SNP59	Exon 6	[AA : AC]	0.0423	*Q* > 0.10	2.7
	SNP70	Intron 9	[AT : AT]	0.0013	0.0490	3.0
Fiber width						
	SNP44	Intron1	[GT : GT]	0.0082	*Q* > 0.10	1.9
	SNP59	Exon 6	[AA : AC]	0.0044	0.0693	2.5
Diameter at breast height (D)						
	SNP89	3'UTR	[CG : CG]	0.0100	*Q* > 0.10	4.0
Tree height (H)						
	SNP51	Intron3	[AC : AC]	2.55E-05	0.0050	3.0
	SNP89	3'UTR	[CG : CG]	0.0351	*Q* > 0.10	2.1
Stem volume(V)						

	SNP18	Promoter	[AT : AT]	0.0093	$Q > 0.10$	1.3
	SNP75	Exon 10	[CT : CT]	0.0088	$Q > 0.10$	5.4

R^2 = percentage of the phenotypic variance explained. *Q*-value = correction for multiple testing [false discovery rate FDR (Q) ≤ 0.10]
